# N-terminal tagging of RNA Polymerase II shapes transcriptomes more than C-terminal alterations

**DOI:** 10.1016/j.isci.2024.109914

**Published:** 2024-05-07

**Authors:** Adam Callan-Sidat, Emmanuel Zewdu, Massimo Cavallaro, Juntai Liu, Daniel Hebenstreit

**Affiliations:** 1 School of Life Sciences, University of Warwick, Coventry CV4 7AL, UK; 2 Warwick Medical School, University of Warwick, Coventry CV4 7AL, UK; 3 School of Computing and Mathematical Sciences, University of Leicester, Leicester LE1 7RH, UK; 4 Department of Physics, University of Warwick, Coventry CV4 7AL, UK

**Keywords:** Molecular biology, Biophysics, Transcriptomics

## Abstract

RNA polymerase II (Pol II) has a C-terminal domain (CTD) that is unstructured, consisting of a large number of heptad repeats, and whose precise function remains unclear. Here, we investigate how altering the CTD’s length and fusing it with protein tags affects transcriptional output on a genome-wide scale in mammalian cells at single-cell resolution. While transcription generally appears to occur in burst-like fashion, where RNA is predominantly made during short bursts of activity that are interspersed with periods of transcriptional silence, the CTD’s role in shaping these dynamics seems gene-dependent; global patterns of bursting appear mostly robust to CTD alterations. Introducing protein tags with defined structures to the N terminus cause transcriptome-wide effects, however. We find the type of tag to dominate characteristics of the resulting transcriptomes. This is possibly due to Pol II-interacting factors, including non-coding RNAs, whose expression correlates with the tags. Proteins involved in liquid-liquid phase separation appear prominently.

## Highlights

• ScRNA-seq datasets for cells expressing CTD-mutant RNA polymerase II (Pol II)
• Transcriptional bursting is robust to Pol II CTD alterations
• N-terminal- rather than CTD-modifications of Pol II shape transcriptomes
• Altered expression of ncRNAs and LLPS-related factors due to N-terminal Pol II tags

## Graphical abstract

**Figure:**
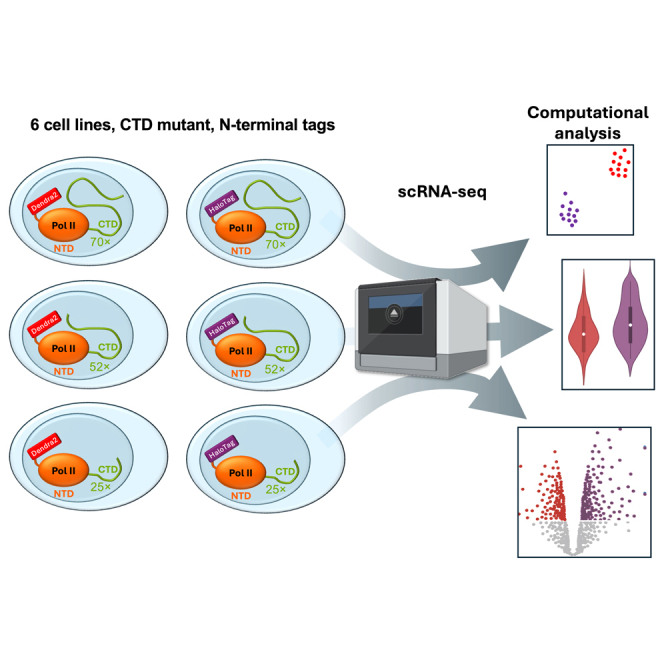

## Introduction

RNA polymerase II (Pol II) transcribes the bulk of eukaryotic genes, including protein coding mRNAs and a variety of non-coding RNAs.^1^ The largest of its twelve subunits, RPB1, is mainly responsible for the enzyme’s catalytic activity. RPB1 has a long, tail-like C-terminal domain (CTD), that consists of multiple repeats of the consensus heptapeptide tyrosine–serine–proline–threonine–serine–proline–serine (Y_1_S_2_P_3_T_4_S_5_P_6_S_7_)^2^ and does not appear to assume a fixed structure.

The presence of CTDs with similar sequence composition and dozens of heptad repeats in very diverse species suggests a fundamental role for it in the transcriptional machinery. *Saccharomyces cerevisiae* and *Schizosaccharomyces pombe* have CTDs with 26 and 29 repeats, respectively, which closely follow the consensus sequence.^2^ The CTD of Drosophila is longer, at 42 repeats, although only two of it match the consensus.^3^ In mammals, the CTD has 52 heptad repeats, of which 21 correspond to the consensus while the rest do not.

Residues within the heptad repeats can become post-translationally modified in patterns that appear linked to the transcriptional stage; S_2_ and S_5_ phosphorylation are arguably the most important ones, accounting for 75% of the total phospho-peptide signal recorded by mass spectrometric measurements.^4^ These two are found to relate to 3′ and 5′ processes, respectively, as mirrored by their intragenic distributions according to ChIP(-seq) data.^2^ On the other hand, the earliest stage of the transcriptional cycle, the assembly of the pre-initiation complex, requires an unphosphorylated CTD, suggesting a dynamical turnover of these modifications.^5^

The post-initiation- and 3′ processes that the CTD and its modifications are involved in include pre-mRNA processing, specifically splicing, and have been summarized in detail in many reviews over the years.^2^^,^^6^^,^^7^^,^^8^^,^^9^^,^^10^^,^^11^ These insights and understanding of its 5′ related functions have been enriched recently by recognizing the CTD’s ability to cluster and form condensates, often described as undergoing liquid-liquid phase separation (LLPS);^12^ this phenomenon has gained prominence recently as a way to explain membrane-less organelles, such as the nucleolus, and appears involved in clustering of transcriptional activators to promote gene expression.^13^^,^^14^^,^^15^ LLPS is thought to be favored by intrinsically disordered regions (IDRs) on proteins, which probably explains the CTD’s propensity to undergo it. LLPS involving the CTD has been described for oncogenic fusion proteins,^16^ RNA binding factors,^17^ and Pol II itself,^5^^,^^18^^,^^19^^,^^20^^,^^21^ the latter in a CTD-length dependent fashion.^5^ Interestingly, truncation of the CTD caused a delay in Pol II response to external triggers but not the steady-state level of mRNA.^22^ More recently, the phosphorylation status of the CTD has also been proposed to regulate shuttling of Pol II between separate LLPS condensates associated with initiation and splicing, respectively.^23^

It is unclear how these phenomena relate to the dynamics of transcription. Transcription of most genes occurs in burst-like fashion, where RNA is produced in large quantities within short bursts of activity, while the genes remain silent in between.^24^ The mechanistic background of this is uncertain, although many different factors have been demonstrated to modulate the size, frequency, or lengths of the bursts, including transcription factor binding sites,^25^ long range chromatin interactions,^26^ chromosomal location,^27^ promoter architecture,^28^ and others, noticeably also interactions between the 3′ and 5′ ends of genes.^29^

The CTD could be the key to understand how bursting and transcriptional subprocesses connect, given its critical roles throughout the transcriptional cycle. Indeed, a recent study reports on a correlation between CTD length and burst size and burst frequency for a gene engineered to permit live RNA imaging in *S. cerevisiae*.^30^

Here, we report on a genome-wide assessment of the CTD’s influence on transcriptional bursting dynamics. Using single-cell RNA sequencing (scRNA-seq) and a collection of mammalian cell lines engineered to express Pol II variants with varying CTD lengths, we demonstrate small but significant effects of the latter on bursting parameters. The types of small protein tags that are fused to the modified Pol II variants appear to dominate overall transcriptional differences between the cell lines. A number of factors are differentially expressed as a result of both, CTD lengths and tags.

## Results

### Mutant cell lines with varying CTD lengths of RPB1

Recent studies^5^^,^^22^ employed a series of cell lines engineered to constitutively express modified versions of the main Pol II subunit RPB1 in addition to its wildtype version. The six lines are all based on U2OS human osteosarcoma cells and share mutations in the ectopic RPB1 sequence that make it resistant to the toxin *α*-amanitin, which binds wildtype Pol II, preventing nucleotide incorporation and translocation, ultimately triggering the ubiquitination and degradation of the bound RPB1.^31^ Treating cells with the toxin thus allows selective removal of the endogenous RPB1, while the modified, ectopic one remains.

The other modifications on the ectopic RPB1 are as follows and can be summarized as having short, wildtype, and longer CTD versions, respectively, with two different tags each (Figure S1); three of the cell lines contain RPB1 tagged with Dendra2, a 26 kDa green-to-red photoconvertible fluorescent protein derived from the octocoral *Dendronephthya* sp.^32^ The CTD of the mutant RPB1 in cell line Dendra2-RPB1-25R (D25) contains repeats 1–21 and 49–52 of human wild-type (WT) RPB1 CTD. Dendra2-RPB1-52R (D52) contains all 52 repeats. Dendra2-RPB1-70R (D70) contains repeats 1–51 and 38–52.

Another three of the cell lines carry RPB1 tagged with HaloTag, a 33 kDa haloalkane dehalogenase whose binding ligand can be fused to a fluorescent dye or affinity tag.^33^ HaloTag-RPB1-25R (H25) and HaloTag-RPB1-52R (H52) contain the same repeats as D25 and D52, respectively. HaloTag-RPB1-70R (H70) contains repeats 1–47, 42–47, and 38–52, of wildtype RPB1. It also contains two other heptad repeats (one between each of the three aforementioned repeat stretches) with the amino acid sequence YSPTSPT, which is the same as wildtype CTD repeats 41, 43, 46, 48, and 51. However, their nucleotide sequences do not match any of the wildtype repeats; the first (repeat 48 in the mutant) has the same nucleotide sequence as CTD 41 of wildtype RPB1 from *Mus musculus*, while the second (repeat 55 in the mutant) does not have a 100% match with a repeat from known species’ sequences. It should be further noted that whilst H70 contains 70 heptad repeats as one would expect from its name, D70 only contains 66 (Figure S1). It is still referred to as D70 in this work, to simplify designation and be consistent with the literature.^5^

All tags are N-terminally linked, with short linker sequences containing a TEV protease recognition motif. The last four repeats of the CTD are identical in all the cell lines, along with the ISPDDSDEEN end sequence (Figure S1), which is likely to facilitate normal splicing, 3′ processing and capping in all cell lines, as these mechanisms are dependent on these C-terminal parts of the CTD.^34^

### Preparation of scRNA-seq data

To investigate the effect of RPB1 CTD length on transcriptional dynamics, the six mutant U2OS cell lines were grown in *α*-amanitin to degrade endogenous RPB1, then RNA of a total of 21,022 cells (Table S1) was sequenced at single-cell resolution using a droplet-based system.

The overall quality of the resulting data was good and we further subjected the samples to data curation following established protocols.^35^ The distributions of the number of genes detected in each cell have long lower tails (Figure S2), prompting us to discard data corresponding to cells with 1,250 or less detected genes since these were likely dead, dying, or broken. We further excluded cells whose molecules were more than 25% mitochondrial (Figures S2B and S2C) since this is indicative of cell damage.^36^ Setting these two threshold parameters removed 526 cells.

### Pol II expression levels differ across cell lines

**Figure 1:**
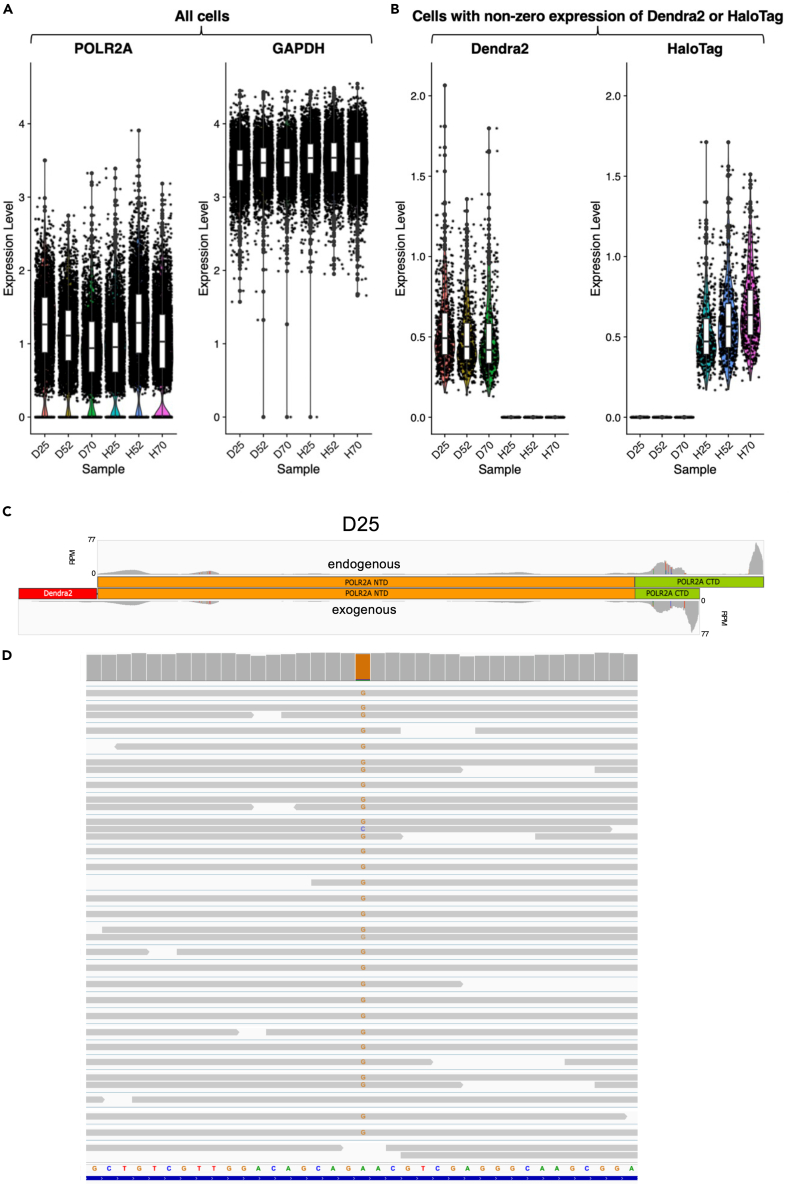
Expression of RPB1 (A) the expression level (normalized and log-transformed UMI count) of POLR2A and GAPDH in all QC-passed cells, and (B) of Dendra2 and HaloTag in QC-passed cells with non-zero expression of either tag. Black points represent individual cells from six U2OS cell lines: Dendra2-RPB1-25R (D25), Dendra2-RPB1-52R (D52), Dendra2-RPB1-70R (D70), HaloTag-RPB1-25R (H25), HaloTag-RPB1-52R (H52), and HaloTag-RPB1-70R (H70). (C) read coverages at the endo- and exogenous reference sequences for the POL2RA mRNA are shown for Dendra2-RPB1-25R (D25) as an example. The other cell lines are shown in Figure S3. Note that the mappings/peaks are not always unique within and across the two. RPM, reads per million total reads. Coverage is computed in 25 bp bins. (D) Integrative Genomics Viewer (IGV) view of reads at the region containing the *a*-amanitin resistance mutation (the central ‘G’ in the view) of the D25 cell line. Most reads have the mutation as indicated. The boxplots in A and B indicate Median (central line inside box), 25% and 75% quartiles (lower and upper edge of box, respectively), 1.5 × inter-quartile range (whiskers). See also Figures S1–S3, Tables S1 and S2, and Data S1.

RPB1 is encoded by the gene POLR2A (RNA Polymerase II Subunit A). Comparing POL2RA reads to those mapping to the housekeeping gene GAPDH suggest that, whilst GAPDH expression appears similar between samples, POLR2A levels are visually different (Figure 1A). Most of these comparisons are statistically significant (Table S2).

The repetitive nature of the endogenous RPB1’s CTD, in addition to sharing much of the sequence with the exogenous CTD, plus the mutants having the identical stretches of repeats several times, makes distinguishing reads mapping to the two versions of Pol II challenging. Accepting a certain fraction of ambiguous mappings, we prepared custom reference sequences (Data S1) for the various RPB1 versions which we could map reads to and compare coverages side-by-side. The resulting plots reveal 3′-heavy mappings and clear detection of the tags, along with co-expression of endogenous and exogenous RPB1, although the latter is expressed more strongly (Figure 1C, D25 shown as example; the other coverages are shown in Figure S3). In agreement with this, we found the position corresponding to the *α*-amanitin resistance mutation (N792D) showing the mutant ‘G’ 20- to 30-times more frequent than the wildype ‘A’ in all cell lines (Figure 1D; D25 shown as example). It is also noteworthy that both the endo- and exogenous RPB1 feature a number of silent mutations compared to the GRCh38 reference genome. For example, one of the instances of repeat 51 in D70 ends in ‘TACA’ instead of ‘CACC’.

**Figure 2:**
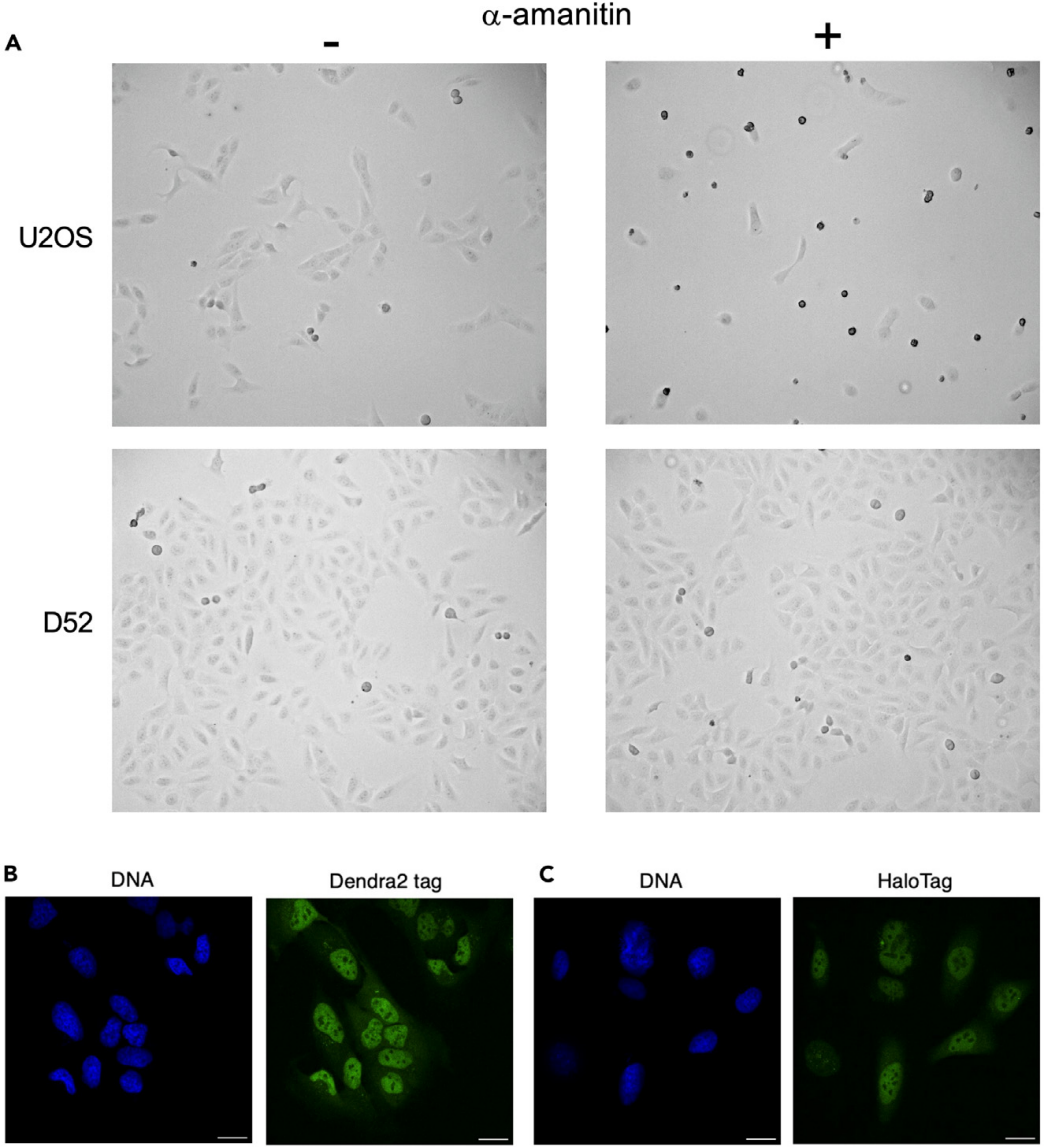
Characterization of cell lines (A) Cells expressing the mutant RPB1 (D52 as example) are viable in *α*-amanitin-containing medium (5 μg/mL) while control U2OS cells are not. The other cell lines are shown in Figure S4. (B) Robust expression of Dendra2-tagged mutant RPB1 in the cell lines. U2OS Dendra2-RPB1-52R (D52) cells stained for DNA (Hoechst 33342, blue). The fluorescent Dendra2 protein is visible in the green channel. (C) Robust expression of Halo-tagged mutant RPB1 in the cell lines. U2OS HaloTag-RPB1-52R (H52) cells stained with Hoechst 33342 for DNA (blue), and Oregon Green HaloTag ligand to detect HaloTag in the green channel. Scale bars = 20 μm. See also Figures S4 and S5.

By considering reads mapping to the tags only, we can assess expression of the ectopic RPB1 constructs exclusively, which is the approach we will use throughout the rest of the manuscript. No Dendra2 specific reads were detected in the HaloTag cell lines and vice versa (Figure 1B). However, tag expression in the correct cell lines was not high either; only 1,647 (8%) of the total 20,416 cells show non-zero Dendra2 expression, and 1,456 (7%) show non-zero HaloTag expression. It should be noted that the RPB1 mRNA is significantly longer than either of the tags’, which might account for some of the difference in detected tag and RPB1 expression. Also, the cell lines are clearly *α*-amanitin resistant (Figures 2A and S4), Dendra2 is detectable by imaging (Figure 2B), and so is the HaloTag upon adding its fluorescently labeled ligand (Figure 2C). Western blotting confirms these expression profiles (Figure S5). Some minimal persisting endogenous RPB1 protein cannot be completely ruled out, though.

Despite low read counts for the tags, some expression differences among the different CTD mutants remain. H70 express significantly more ectopic HaloTag mRNA than the others (Figure 1B). This contrasts with the findings of,^5^ who report lower RPB1 protein expression for the 70-repeat cell lines than the others in both tag versions. RNA and protein levels are not directly comparable, though, which might explain the different findings.

### Estimation of transcriptional bursting parameters

We proceeded to characterize transcriptional bursting in the cell lines. A Markov chain Monte Carlo (MCMC) sampling approach was employed to fit a negative-binomial model for gene expression^29^ to the data of all QC-passed cells, and estimate the mean expression (*μ*), squared coefficient of variation (CV^2^), and burst frequency (k_on_) of each gene. The shape of the mRNA distributions across cells varies in characteristics ways depending on these and other parameters (see below; Figure S6), which in turn allows their reliable estimation from the data. This approach of using single-cell RNA distributions at a single time point as provided by assays such as smFISH or scRNA-seq to infer transcriptional dynamics has been used successfully in many cases,^29^ as transcriptional fluctuations over time introduce heterogeneity across a cell population.^37^

**Figure 3:**
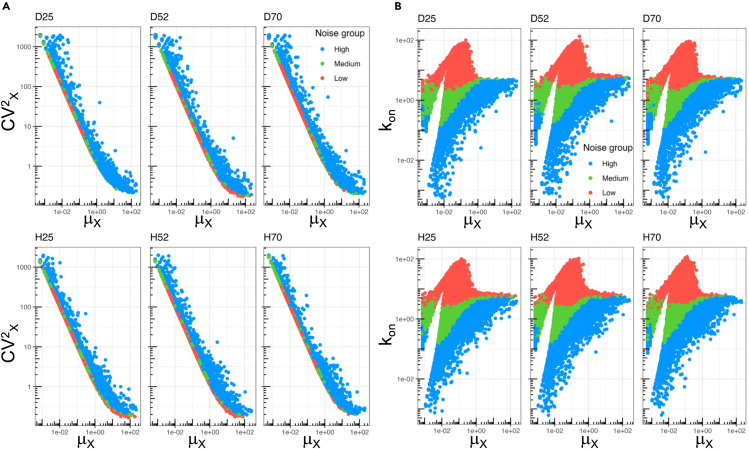
Transcriptional noise (A) Mean expression (*μ*_X_) vs. CV^2^_X_ colored by noise group. The six samples are the mutant U2OS cell lines: Dendra2-RPB1-25R (D25), Dendra2-RPB1-52R (D52), Dendra2-RPB1-70R (D70), HaloTag-RPB1-25R (H25), HaloTag-RPB1-52R (H52), and HaloTag-RPB1-70R (H70). (B) as A, for Mean expression (*μ*_X_) vs. k_on_. See also Figures S6 and S7.

The CV^2^ of the mRNA abundance is typically used as a measure for the transcriptional ‘noise’ and was found to decrease as the estimated mean expression level increased (Figure 3A), an observation seen before in other systems (e.g.,^29^^,^^38^^,^^39^^,^^40^).

To study noise in more detail, we introduced a separate measure for it that removes the influence of expression level and corresponds to the vertical distance *ν* of each gene’s CV^2^ to the curve CV^2^_X_ = 1/*μ*_X_ + 1/${\bar{k}}_{\text{on}}$ in logarithmic scale, where ${\bar{k}}_{\text{on}}$ is the burst frequency averaged over all genes. This curve describes the trend seen when plotting CV^2^ vs. mean (Figure 3A) and can be derived from the underlying negative binomial model.^29^

We used *ν* to define three equal groups of noise level (low, medium, or high) to facilitate additional analyses (Figure 3A). Plotting these groups against burst frequency (k_on_) confirms the expected inverse relation between k_on_ and noise – the more frequent the bursts, the more ‘regular’ and less noisy transcription becomes (Figure 3B). We also calculated the average burst size *α*/k_off_ (*α* is the transcription rate during bursts, k_off_ is the rate to switch to the ‘off’ state from the bursting state),^29^ which shows the opposite behavior in terms of noise (Figure S7), again in accordance with theory. The higher burst size among the more highly expressed genes explains why these tend to be in the ‘high’ noise group for a given k_on_. This is also confirmed by our analyses of the expected relations among parameters: at constant k_on_, the expression level lowers with decreasing burst size (Figure S6, left to right).

**Figure 4:**
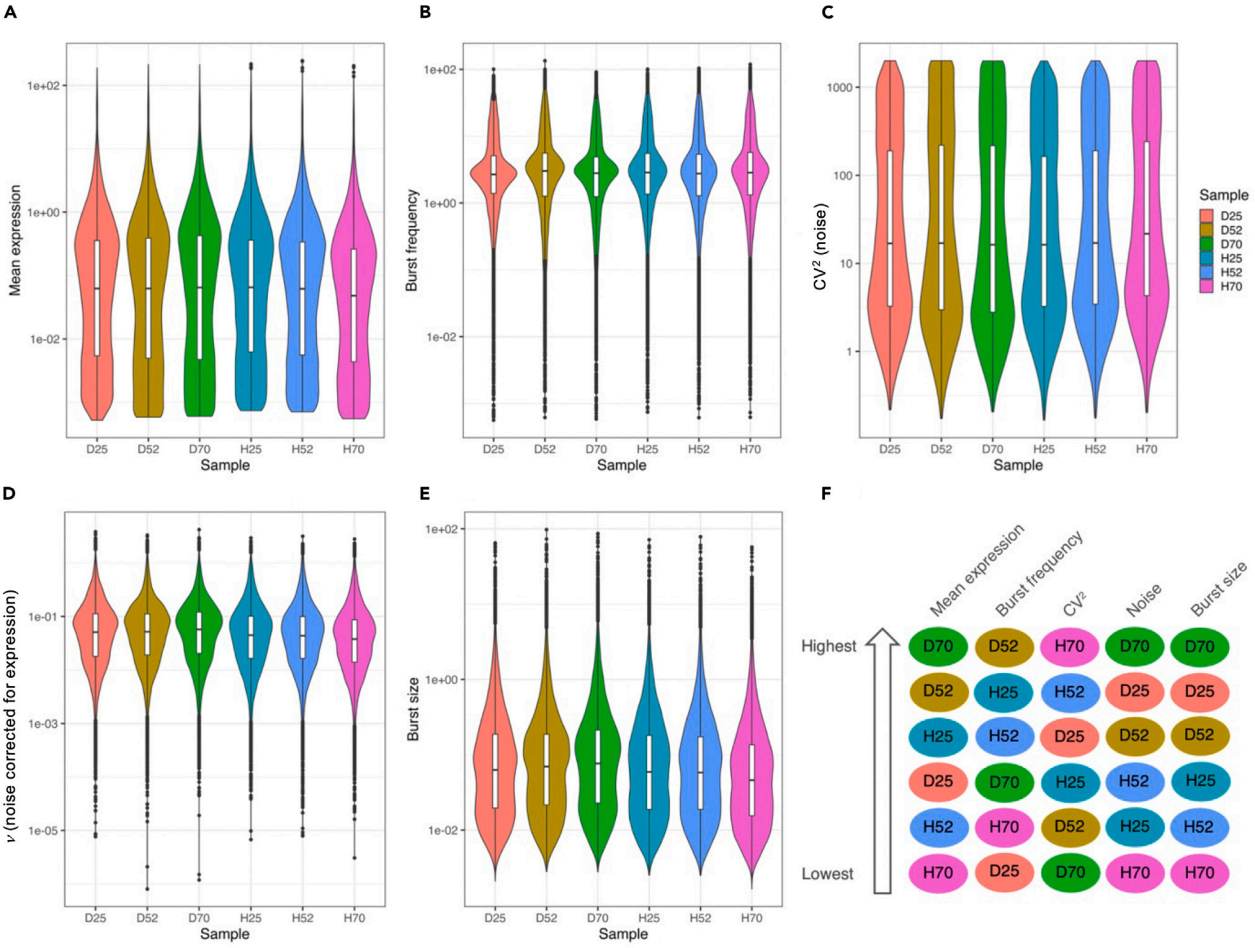
Kinetic parameters (A–E) Violin plots with logarithmic y axes and boxplot overlays comparing kinetic parameters estimated from the scRNA-seq data of the six mutant U2OS cell lines (D25/52/70, H25/52/70) as indicated in the plots. The boxplots indicate Median (central line inside box), 25% and 75% quartiles (lower and upper edge of box, respectively), 1.5 × inter-quartile range (whiskers). (F) The fifteen pairwise combinations of the six samples (cell lines) were used to compare the kinetic parameters of each gene individually. The sample with the highest number of genes with the highest parameter in each pair was recorded, and the fifteen results were compiled to order the samples. See also Figures S8–S11 and S20 and Tables S3–S12.

We next compared the six cell lines with regard to all five quantities (mean, burst frequency, CV^2^, *ν*, burst size). Surprisingly, all six have very similar distributions of these (Figures 4A–4E). The fifteen pairwise combinations of the six samples were used to compare the parameters of each gene individually. While almost all sample pairs (for all parameters) were shown to be statistically different from one another with very low *p* values (Tables S3–S7), this is presumably due to the high interdependency of gene expression levels, since tens of thousands of correlated measurements are being tested; the differences in actual values are small, though.

To present the results more compactly, we recorded the sample with the highest number of genes with the highest parameter value in each pair, and the fifteen results were compiled to order the samples. Mean expression and CV^2^ are the exact inverse of each other, and burst size and noise are almost exactly positively correlated (Figure 4F).

Repeating the analysis using only the 3,103 cells that have non-zero expression of either tag gave similar results (Figures S8A–S8E; Tables S8–S12). When testing the order of the parameters as above, mean expression, CV^2^, and *ν* maintained ordering of the samples, while the order of the other three parameters differed (Figure S8F). The lower number of cells and the similarity of the distributions probably means that both, differences to the results using all cells, but also actual differences between the cell lines, are rather small.

Since the ectopic Pol II are expressed at slightly different levels in the cell lines, as stated in the previous section, we also wanted to test if some of the differences among the transcriptional dynamics we observe could be ascribed to this. To this end, we built on a previously published model^29^ that can relate the amount of available Pol II to kinetic parameters. Plotting the latter as a function of varying Pol II concentration shows modest effects, with mean and noise largely unaffected, while burst size and k_on_ in- and decrease with Pol II to a certain degree, respectively (Figure S9).

We examined this in our data and plotted the parameter estimates against the (overall) Pol II expression levels in our cell lines. While there is some support for the reciprocal scaling of burst size and k_on_ as described above when the ‘70’ cells are considered, these trends are not clear at all when the other cell lines are taken into account (Figure S10). We conclude that, although we cannot exclude Pol II levels affecting our bursting estimates, the contributions of this factor appeared to be minor at best.

As transcription in our cell lines is carried out by mutant Pol II versions, it seemed sensible to also compare our bursting estimates with those of wildtype Pol II. For this purpose, we considered bursting data we had obtained from A549 cells in one of our earlier studies.^29^ The distributions of parameter values are similar, but some differences emerge; the mean expression levels of the A549 cells are slightly lower than the U2OS’ which might be due to reduced burst-sizes in the former, while k_on_ values show the opposite behavior (Figure S11). Variations in noise (Figure S11) probably stem from these differences.

While our cell lines and the A549 cells’ transcriptional dynamics thus seem comparable overall, a further exploration of their differences might be an interesting subject for a follow-up study, potentially including other cell lines as well; it seems possible that, aside from the altered RPB1 sequences, epigenetic effects and other mechanisms affect bursting kinetics in ways that are characteristic for different cell types.

### Transcriptome-wide analyses reveal cell line differences

**Figure 5:**
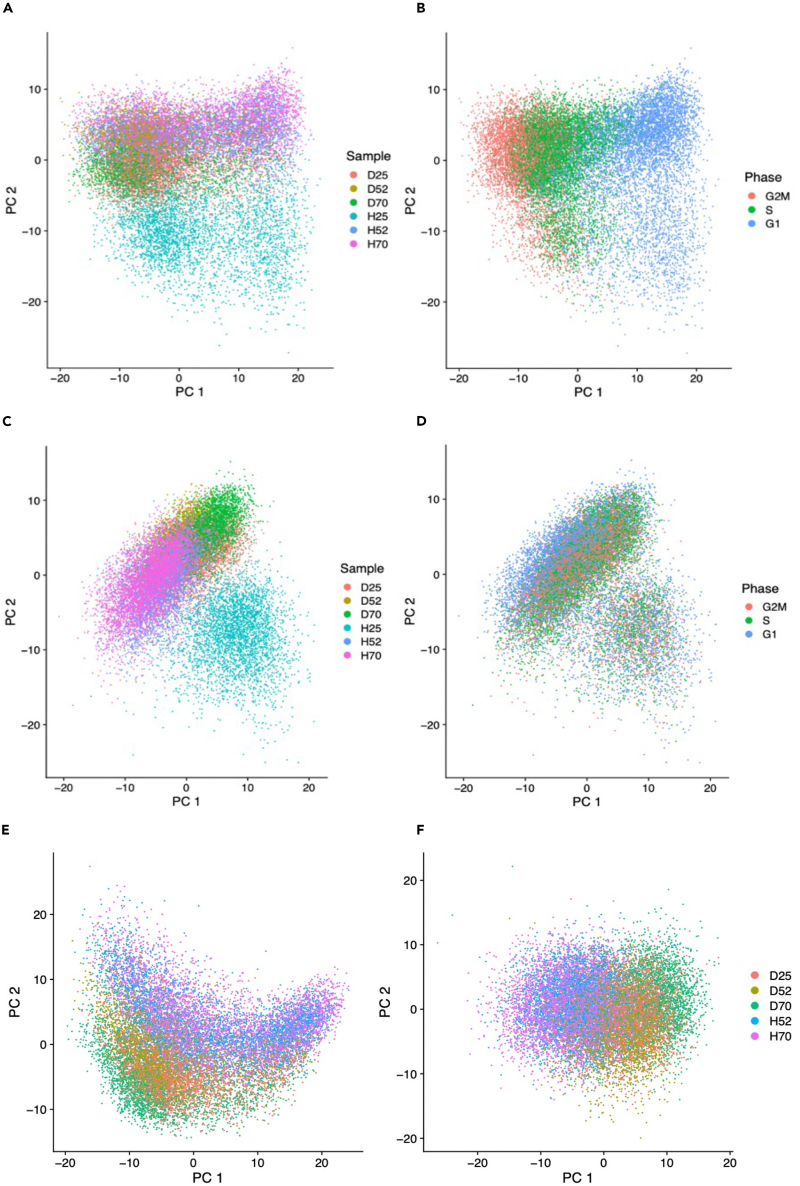
PCA plots before and after cell cycle phase regression The top two principal components representing scRNA-seq data before (A, B) and after (C, D) regressing for cell cycle phase. Samples are the mutant U2OS cell lines: Dendra2-RPB1-25R (D25), Dendra2-RPB1-52R (D52), Dendra2-RPB1-70R (D70), HaloTag-RPB1-25R (H25), HaloTag-RPB1-52R (H52), and HaloTag-RPB1-70R (H70). (A) PCA on variable genes, colored by sample. (B) PCA on variable genes, colored by cell cycle phase. (C) PCA on variable genes after regressing for cell cycle phase, colored by sample. (D) PCA on variable genes after regressing for cell cycle phase, colored by cell cycle phase. (E and F) PCA plots after H25 removal, before (E) and after (F) cell cycle phase regression. See also Figures S12–S16 and Table S13.

To capture overall differences among the cell lines’ transcriptomes, we carried out a principal component analysis (PCA) on the 2,000 most variable genes (see STAR Methods). The first two components capture 36% of the total variance (Figure S12). Plotting these revealed H25 to be strongly separated from the other five samples, while the latter overlapped heavily (Figure 5A). PCs 3–12 appeared to not separate the cell lines either (Figure S13), and results were conserved when selecting cells with non-zero tag expression only (Figure S14).

Comparing the top genes of the significant PCs to S and G2/M phase markers revealed that PC1, PC3, and PC5, were dominated by cell cycle genes (Figure S15; Table S13). We thus gave each cell a score dependent on its expression of S phase and G2/M phase markers and assigned its most likely cell cycle phase,^41^ which showed that cells were separating by their cell cycle stage in the plot of the first two principal components (Figure 5B), potentially masking cell line differences. To remove this effect, we regressed the variation due to each cell’s stage in the cell cycle against each gene.^42^ Repeating the PCA on the 2,000 most variable genes (Figures 5C and S16) showed that though this correction was successful (Figure 5D), H25 is still separate from the other five samples. This is not unexpected since there was a similar proportion of cell cycle phases between the samples (Figures 5A and 5B).

As the strong separation between H25 and the other five samples could be masking other differences, H25 was removed and the above analysis repeated. However, the five samples appear to cluster by tag, rather than RPB1 tail length (Figures 5E and 5F).

**Figure 6:**
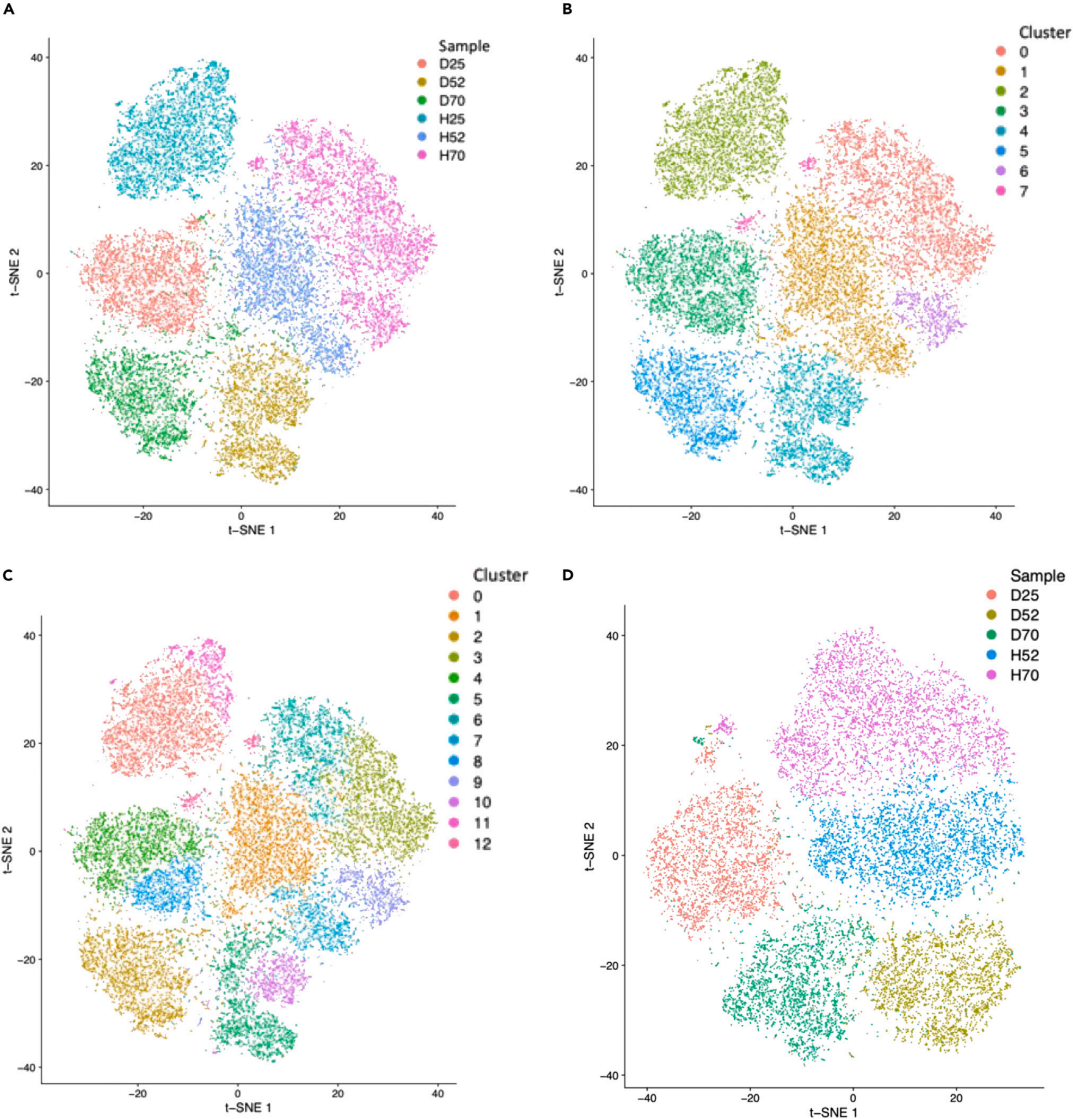
Nonlinear dimensionality reduction plots QC-passed cells after cell cycle phase regression from six mutant U2OS cell lines were used: Dendra2-RPB1-25R (D25), Dendra2-RPB1-52R (D52), Dendra2-RPB1-70R (D70), HaloTag-RPB1-25R (H25), HaloTag-RPB1-52R (H52), and HaloTag-RPB1-70R (H70). (A) t-SNE plot colored by sample. (B) t-SNE plot colored by cluster, after clustering with a resolution parameter of 0.4. (C) t-SNE plot colored by cluster, after clustering with a resolution parameter of 1.0. (D) t-SNE plot colored by sample, excluding H25. See also Figure S17.

As two additional, nonlinear dimensionality reduction approaches, we subjected the data to t-distributed stochastic neighbor embedding (t-SNE^43^) and uniform manifold approximation and projection (UMAP^44^) based on the cell cycle corrected PCA projections. Both approaches (Figures 6A–6C and S17A–S17C) show that the generated clusters are essentially divided by sample, and that H25 is the most separated from the five other cell lines as before, although the separation is more pronounced in the UMAP plot (Figures S17A–S17C). Except for D70, t-SNE 1 and UMAP 1 appear to separate the cells by tail length, although this is tenuous (Figures 6A and S17A); removing H25 and repeating the analysis shows a slight tendency for the samples to group by tag type more than tail length once again (Figures 6D and S17D).

### CTD-length dependent expression of individual genes

**Figure 7:**
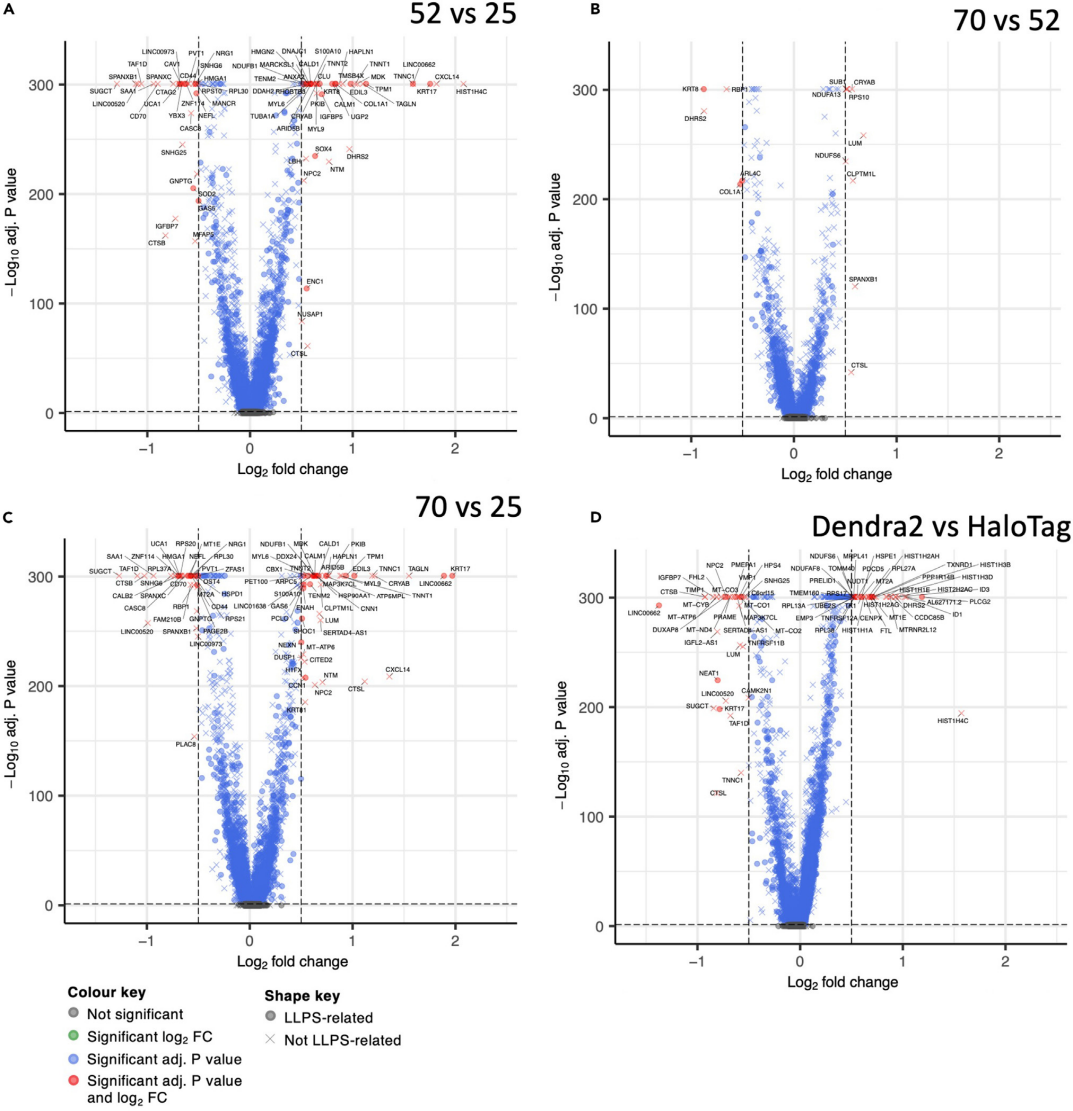
Volcano plots of differential gene expression Different combinations of the cell lines are shown as indicated. A positive log_2_ fold change indicates the gene is more highly expressed in the cell line with the longer CTD (A–C) or in the Dendra2 cells (D). *p* values adjusted using the Bonferroni correction. Color and shape of the symbols relate to significance and LLPS-relation, respectively, as indicated. See also Figure S18 and Tables S14 and S15.

As another approach to tease out transcriptional differences relating to CTD lengths, we grouped datasets by the latter and carried out pairwise tests for differential gene expression (D52 & H52 vs*.* D25 & H25; D70 & H70 vs*.* D52 & H52; D70 & H70 vs*.* D25 & H25). Using combined log_2_ foldchange (≥|½|) and *p* value (<0.05, Bonferroni corrected) thresholds, moderate numbers of significantly differentially expressed genes emerged (Table S14). Among these, two genes are worth highlighting; CRYAB and CTSL were more positively expressed in the cell line with the longer RPB1 CTD in all three comparisons as shown in Volcano plots (Figures 7A–7C).

CRYAB encodes αB-crystallin (CryAB), also known as heat shock protein B5 (HspB5), which localises to nuclear speckles^45^^,^^46^ upon phosphorylation.^47^^,^^48^ CryAB acts as a molecular chaperone that suppresses the aggregation of various proteins.^49^^,^^50^ CryAB is upregulated in U2OS cells that lack the ataxia telangiectasia mutated (ATM) kinase, or that have been treated with the topoisomerase inhibitor camptothecin, two conditions which cause the widespread aggregation of proteins prone to LLPS.^51^^,^^52^ Overexpressing CryAB in ATM knockout and camptothecin-treated U2OS cells rescues protein aggregation back down to the untreated WT level.^52^ Other heat shock protein chaperones have been shown to monitor condensates^53^ and prevent LLPS from progressing into abnormal protein aggregates.^54^

CTSL encodes cathepsin L, a protease that clears toxic protein aggregates.^55^^,^^56^ The upregulation of CRYAB and CTSL in cells expressing RPB1 with a longer CTD could indicate that the longer CTD causes increased LLPS *in vivo*, resulting in a compensatory increase in CRYAB and CTSL.

To analyze more systematically the potential association of differentially expressed genes with LLPS, we used two databases (PhaSepDB & RPS) to classify genes into LLPS-related ones and others. We indicate this classification in the volcano plots (Figure 7) and plot proportions of LLPS-related genes as bar-charts (Figure S18A), with absolute numbers shown in Table S15. The results demonstrate a solid but not significant (Fisher’s exact test, Bonferroni-corrected) enrichment for LLPS-related genes among those upregulated in samples with longer CTD tails, expect the 70 vs. 52 comparison, but these are only nine genes (and not significant either).

### Pol II tags affect gene expression in characteristic ways

Since overall transcriptional differences between the cell lines appeared to mostly relate to the type of tag added to RPB1 (Figures 5, 6A–6D, and S17), we sought to understand this effect better by looking at individual genes as well. To this end, we grouped the datasets by tag and carried out tests for differential expression as before (D25 & D52 & D70 vs*.* H25 & H52 & H70). This again produced a moderate number of significantly differentially expressed genes (Table S14). Many of these appeared to have housekeeping roles, including many histone, mitochondrial, and ribosomal genes (Figure 7D) and do not seem to be enriched for LLPS-related genes (Figure S18A). Interestingly, CTSL makes an appearance again, being favored to be expressed by the Halo-tagged cell lines. Another interesting gene more highly expressed in the latter is nuclear-enriched abundant transcript 1 (NEAT1), which we describe in more detail as part of our next analysis below.

To quantify tag-dependent expression of individual genes in an improved way, we exploited the single cell resolution of our data and identified genes based on their co-expression with the tags, while accepting that this will also yield many genes co-expressed with Pol II in general.

We calculated the Pearson correlation coefficients between each gene’s and the exogenous tags’ (Dendra2 or HaloTag) expression levels. After removing genes with no expression in any cells, we obtained 3,213 genes with significant correlations (*p* < 0.05, BH corrected) for Dendra2, and 2,740 for the HaloTag samples.

**Figure 8:**
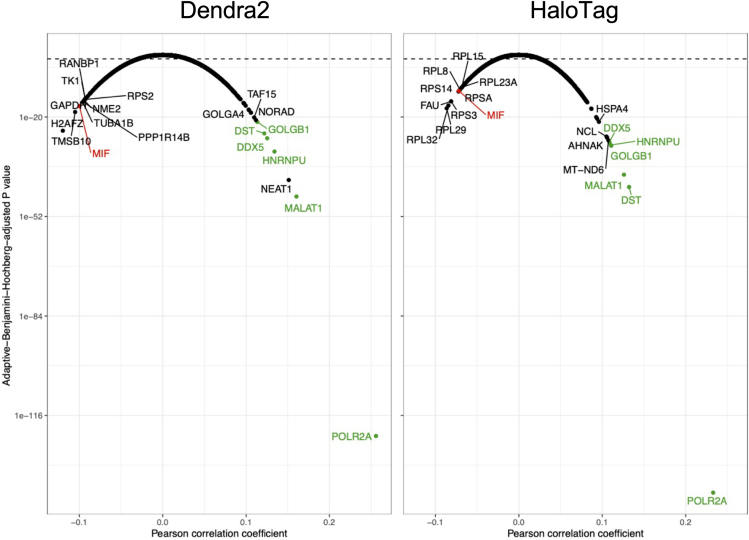
Correlation between exogenous tag expression and gene expression The linear relationships between tag (Dendra2 and HaloTag, as indicated) expression and the expression of all other genes were evaluated using scRNA-seq data from the mutant U2OS cell lines D25, D52, and D70. The Pearson correlation coefficient was plotted against the adaptive Benjamini-Hochberg-adjusted *p* value. The top ten genes whose expression most negatively correlated with tag expression were labeled, and those in the top ten of both tags were colored red. The same was done with the most positively correlated genes, with the common genes colored green. The horizontal dashed line is at *p* value = 0.05. See also Figure S19.

Out of the top ten genes whose expression was most positively correlated with tag expression for each of the two tags, six were present in both lists (Figure 8). The most positively correlated by far was POLR2A, which is to be expected since it encodes RPB1, which (in its various mutated forms) is the other half of the tagged fusion protein. Of course, transcripts encoding potentially remaining, untagged endogenous RPB1 are also included in the measured POLR2A expression level.

We now also see a strong enrichment of LLPS-related genes among those positively correlated with the tags (Figure S18B), some of which are discussed in more detail below, while those negatively correlated are strongly depleted of the former (all *p* < 10^−20^, Fisher’s exact test, Bonferroni-corrected).

Metastasis-associated lung adenocarcinoma transcript 1 (MALAT1) was also in the top ten of both tags. Another name for MALAT1 is nuclear-enriched abundant transcript 2 (NEAT2), and NEAT1 is in the top ten of Dendra2 samples. Both are long non-coding RNAs (lncRNAs) that bind active chromatin sites,^57^ and localize to granular structures in the nucleus where they act as scaffolds.^58^ NEAT1 localizes to nuclear paraspeckles and is essential for their formation,^59^ phase separation,^60^ and structure.^61^ It interacts with the Integrator complex (Barra et al., 2020), which binds to the CTD of Pol II.^62^ NEAT1 also facilitates the phosphorylation of serine 2 on the CTD of Pol II.^63^ NEAT2 localizes to the polyadenylated component of nuclear speckles^64^ but is not essential to their formation^64^ or structure.^65^ NEAT2 regulates splicing by interacting with spliceosomal proteins.^66^^,^^67^

Heterogeneous Nuclear Ribonucleoprotein U (HNRNPU), also known as scaffold attachment factor A (SAF-A), regulates RNA splicing and chromatin structure.^68^^,^^69^ Its NTD binds to DNA, and its CTD binds to RNA,^70^ including the aforementioned lncRNAs NEAT1 and NEAT2.^71^ Interestingly, it was shown to interfere with Pol II elongation.^70^

Dystonin (DST, also known as BPAG1) encodes a cytoskeletal linker protein whose functions include preventing the accumulation and aggregation of neurofilaments, vacuoles, and membrane-bound organelles.^72^^,^^73^ Despite being a large structural protein responsible for maintaining cytoarchitecture integrity, DST is capable of localizing to the nucleus.^74^

Golgin subfamily B member 1 (GOLGB1) is in both top ten lists, and another golgin-encoding gene, Golgin subfamily A member 4 (GOLGA4), is in the top ten of Dendra2 samples. GOLGB1 and GOLGA4 both encode long coiled-coil proteins that attach to the Golgi apparatus via their CTDs,^75^ whilst their N-terminal domains (NTDs) capture transport carriers.^76^

DEAD-Box Helicase 5 (DDX5) encodes an ATP-dependent RNA helicase. Its NTD binds to Pol II,^77^^,^^78^ recruiting the polymerase to promoters,^79^ and its CTD is an IDR which facilitates its LLPS.^80^

Three of the five genes (ignoring POLR2A) that are in the top ten lists of both tags interact with Pol II directly or indirectly, and are involved in phase separation (MALAT1, HNRNPU, and DDX5). Expression of the tagged mutant protein could have caused these proteins and lncRNAs to interact, phase separate, and be sequestered. This could have induced their increased transcription through regulatory feedback networks.

The one gene that was in the top ten most negatively correlated genes for both tags was macrophage migration inhibitory factor (MIF), a pleiotropic pro-inflammatory cytokine involved in osteosarcoma progression.^81^ Since U2OS cells were isolated from an osteosarcoma, they express MIF at a higher level than normal osteoblast cells.^82^ The tags might interfere with its expression.

Finally, we repeated the correlation analysis for individual samples instead of pooling by tag type, with similar results (Figure S19).

## Discussion

Our work presents a genome-wide analysis of transcription-related characteristics of six cell lines engineered to express tagged mutants of the main Pol II subunit RPB1. Interestingly, we found only minor differences in bursting parameters; though statistically significant, burst size and burst frequency, along with mean expression and noise, differ by relatively small amounts, and are not following an obvious trend when CTD lengths are considered. This is somewhat contrasting with findings in *S. cerevisiae*, where a scaling of burst size and frequency with CTD length was observed.^30^ It should be noted though that this was carried out on a single gene that might not be representative of the transcriptome. Indeed, individual genes with these properties can be identified in our analyses of the human cell lines, too (three examples are shown in Figure S20). On the other hand, given the evolutionary distance of budding yeast and humans, as also illustrated by fundamental differences such as the absence of polymerase ‘pausing’ in the former,^83^ make it plausible that the CTD dependence of bursting does differ.

Another recent study^22^ examined transcription in two of the cell lines we used here, H52 and H25. Even though H25 is the sample we identified as an outlier here - potentially amplifying differences to H52 - the authors report a lack of CTD-length related global transcriptional changes, confirming our findings of only minor differences between the lines. The study does describe effects on Pol II pausing and various other impacts on gene regulation, but does not explore bursting or other aspects that would have required single cell resolution. It will be interesting to see if the other cell lines show similar Pol II pausing phenotypes.

Our findings overall suggest that the Halo and Dendra2 tags have bigger effects on transcription than the CTD mutations. While we do identify genes that are differentially expressed in CTD length-dependent fashion, these are not many, and our multivariate analyses also support bigger roles for the tags than the CTD lengths. These observations merit a closer look. Though the tags are (relatively) small at 33 and 26.1 kD, respectively, they have defined structures and potentially exhibit very different biophysical characteristics than the CTD, possibly leading to an altered function of RPB1/Pol II. This might have caused the enriched, LLPS-related proteins and transcripts (lncRNAs) we observe to correlate with the tag expression to interact, phase separate, and be sequestered. This could have reduced their capacity to fulfill their cellular functions, and so induced their increased transcription through regulatory feedback networks. Further experiments and analysis will be required to test this theory, though.

### Limitations of the study

Our study was based on a number of engineered cell lines. Spurious effects of the engineering work, along with genetic drift as a result of extensive culturing might have contributed to the differences we observed between the lines. Alternative N-terminal tags or C-terminal modifications also might elicit distinct effects from the ones we found.

## STAR★Methods

### Key resources table

**Table:** 

REAGENT or RESOURCE	SOURCE	IDENTIFIER
**Antibodies**		

anti-Pol II, clone F-12	Santa Cruz	sc-55492; RRID: AB_630203
anti-Dendra2, clone OTI1G6	ThermoFisher	TA180094; RRID: AB_2622288
Anti-HaloTag	Promega	G9211; RRID: AB_2688011
anti-GAPDH, clone 1E6D9	ThermoFisher	60004-1-IG; RRID: AB_2107436
*m*-IgGK BP-HRP	Santa Cruz	sc-516102; RRID: AB_2687626

**Chemicals, peptides, and recombinant proteins**		

Oregon Green HaloTag Ligand	Promega	G2802
α-Amanitin	Merck	A2263

**Critical commercial assays**		

Chromium Next GEM Single Cell 3′ GEM Library & Gel Bead Kit v3.1	10x Genomics	PN-1000121
Chromium Next GEM Chip G Single Cell Kit	10x Genomics	PN-1000127
Chromium Dual Index Kit TT Set A (DUAL INDEX)	10x Genomics	PN-1000215

**Deposited data**		

Raw and analyzed scRNA-seq data	This paper	GEO: GSE217243

**Experimental models: Cell lines**		

Human: U2OS HaloTag-RPB1-25R	Boehning M et al.^5^	N/A
Human: U2OS HaloTag-RPB1-52R	Boehning M et al.^5^	N/A
Human: U2OS HaloTag-RPB1-70R	Boehning M et al.^5^	N/A
Human: U2OS Dendra2-RPB1-25R	Boehning M et al.^5^	N/A
Human: U2OS Dendra2-RPB1-52R	Boehning M et al.^5^	N/A
Human: U2OS Dendra2-RPB1-70R	Boehning M et al.^5^	N/A
Human: LLA12 (U2OS HaloTag-RPB1-52R derived)	This paper	N/A

**Software and algorithms**		

bcl2fastq	Illumina	https://emea.support.illumina.com/downloads/bcl2fastq-conversion-software-v2-20.html
Seqtk	CSC – IT Center for Science, Finland	https://github.com/lh3/seqtk
Genomics Cell Ranger 6.1.2 pipeline	10x Genomics	https://www.10xgenomics.com/support/software/cell-ranger/latest
R	R Development Core Team^84^	https://www.r-project.org/
Seurat	Hao Y et al.^42^	https://satijalab.org/seurat/
gLoop MCMC sampler	Cavallaro M et al.^29^	https://github.com/mcavallaro/gLoop

### Resource availability

#### Lead contact

Information and requests for resources and reagents should be directed to and will be fulfilled by the lead contact, Daniel Hebenstreit, d.hebenstreit@warwick.ac.uk.

#### Materials availability

This study did not generate new unique reagents.

#### Data and code availability

• Single-cell RNA-seq data have been deposited at GEO and are publicly available as of the date of publication. Accession numbers are listed in the key resources table. Original western blot images and microscopy data reported in this paper will be shared by the lead contact upon request.
• This paper does not report original code that has not been published before or that is more complex than running basic commands of packages.
• Any additional information required to reanalyze the data reported in this paper is also available from the lead contact upon request.

### Experimental model and study participant details

U2OS-based cells were grown at 37°C and 5% CO_2_, in DMEM supplemented with 10% FBS and were gifted from Boehning M et al. 2018. The control U2OS cells (Figure 2A), termed ‘LLA12’, are derived from the D52 line and have lost the ectopic Rpb1 expression. The cell lines are reported to be of female origin. The scRNA-seq experiments we carried out authenticated identity of the cell lines by confirming presence and expression of the modified Rpb1 constructs.

### Method details

#### Microscopy

For the viability assay (Figures 1C and S4), cells were seeded into a 24-well Corning Costar cell culture plate (Fisher Scientific), incubated in *α*-amanitin for 14 days, and imaged with an Echo Revolution Automated Microscope.

To monitor mutant RPB1 expression (Figures 2B and 2C), cells were grown for 2 days on 35 mm dishes with a 20 mm tissue culture-treated glass center of 0.17 ± 0.01 mm thickness (VWR). Halo-tagged cells were stained with 1μM HaloTag Oregon Green Ligand (Promega), then all cells were stained with 5 μg/mL Hoechst 33342 dye (Thermo Fisher Scientific), both times according to the manufacturers’ instructions. Cells were imaged in Gibco DMEM No Phenol Red (Fisher Scientific) supplemented with 10% FBS, in an imaging chamber maintained at 37°C and 5% CO_2_. Images were acquired with a spinning disk microscope (Nikon Ti-2 Eclipse with Yokogawa CSU-X1 spinning unit and iXon-888 EMCCD detector) and a 40x oil immersion objective (Plan Fluor 40x/1.30 Oil DIC H/N2, Nikon), using 405 and 488 nm lasers to excite Hoechst 33342 (blue channel) and Oregon Green/Dendra2 (green channel), respectively.

#### Cell culture, data generation and processing

The six U2OS cell lines (D25, D52, D70, H25, H52, and H70) were incubated with *α*-amanitin (1 *μ*g/ml) for six days, then harvested at 80% confluence and washed according to the 10X Genomics sample preparation protocol (except 1X PBS with 0.5% BSA was used instead of the suggested 1X PBS with 0.04% BSA). Droplet encapsulation and library preparation was performed by following the 10X Genomics Chromium Single cell 3′ protocol using Chromium Next GEM Single Cell 3′ GEM Library & Gel Bead Kit v3.1, Chromium Next GEM Chip G, and Dual Index Kit TT Set A. The cDNA amplification and sample index PCRs were performed with 12 and 11 cycles, respectively. The libraries were run with 1% PhiX spike-in on a 150 cycle High v2.5 flow cell using the NextSeq 500 system (28 cycles for read 1, 10 cycles for index i7, 10 cycles for index i5, and 90 cycles for read 2). The Illumina base call files were demultiplexed with bcl2fastq conversion software (version 2.20), and the resulting fastq files were randomly subsampled with seqtk (https://github.com/lh3/seqtk) to account for differences in sequencing depth. Subsampling was performed with a random seed of 100, and the fractions of reads to be subsampled using reservoir sampling were defined (D25: 1.000000000, D52: 0.964703320, D70: 0.779511946, H25: 0.654947785, H52: 0.535025616, H70: 0.543084659) since this approach only stores one record in memory. Setting a fixed number of reads to be subsampled was not feasible with the size of this dataset since it requires that number of records to be kept in memory. The subsampled reads were aligned to an edited version of the GRCh38 2020 reference genome containing Dendra2 and HaloTag sequences as features, then filtered and counted (Table S1) with the 10X Genomics Cell Ranger 6.1.2 pipeline.^85^ Separately, we aligned reads to manually constructed sequences of the exogenous and the endogenous POL2RA mRNA sequences, respectively, which took account of the mutations we observed. The data was deposited at gene expression omnibus (GEO, https://www.ncbi.nlm.nih.gov/geo/), accession number: GSE217243.

#### Western blotting

Cells were lysed using RIPA buffer (Pierce) supplemented with Complete Mini EDTA-free protease inhibitor (Roche diagnostics GmbH). Cell lysis was further aided by passing cells through 18-gauge needles attached to 2mL syringes. Diluted Laemmli buffer solution (Bio-Rad), mixed with 0.1M DTT (ThermoFisher Scientific), was mixed with equal amounts of supernatant from centrifuged cell solution for western blotting analysis.

Blotting was done using BIORAD mini-protein TGX precast gels and Trans-Blot Turbo 0.2μm (pore size) Mini Nitrocellulose membranes utilising a BIORAD Trans-Blot Turbo System. Blocking was performed in TBST buffer supplemented with 3% BSA (SIGMA life science). Detection was carried out using SuperSignal West Pico PLUS Chemiluminescent Substrate, followed by imaging with a BIORAD ChemiDoc MP imaging system.

The following primary antibodies were used: anti-Pol II, clone F-12 (Santa Cruz, Santa Cruz sc-55492), anti-Dendra2, clone OTI1G6 (ThermoFisher, TA180094), Anti-HaloTag (Promega, G9211) anti-GAPDH, clone 1E6D9 (ThermoFisher, 60004-1-IG). The following secondary antibodies were utilised during western blotting and imaging: *m*-IgGK BP-HRP (Santa Cruz, sc-516102). Abcam Broad Molecular Weight ladder (ab116028) was used to visualise bands following imaging.

#### Data analysis

A merged Seurat^42^ object was created with the unique molecular identifier (UMI) count matrices, and numerous Seurat functions were used to process and explore the data (all functions in this paragraph are from the Seurat R package). The PercentageFeatureSet function was used to calculate quality metrics, which were visualised with the VlnPlot and FeatureScatter functions. The data were filtered to exclude cells with low numbers of detected genes and high mitochondrial coverages as explained in the main text using the subset function. Gene expression was then normalised in each cell with the NormalizeData function by dividing the counts of each gene by the counts of all genes, then multiplying by a scale factor of 10,000 transcripts per cell, and natural log-transforming. We preceded multivariate analyses by filtering datasets for the most informative genes. To identify the most variable genes while accounting for heteroscedasticity, the FindVariableFeatures function was used to group genes into 20 bins based on their average expression and average variance, and their average variance was normalised within that bin to create a standardised variance *Z* score.^86^ The normalised expression levels of the 2,000 most variable genes were centered around 0 by subtracting the mean expression of each gene. This centered expression was then divided by the standard deviation of the gene, scaling the variance of each gene to 1. Centring and scaling the data with the ScaleData function ensured lowly and highly expressed genes contributed equally to downstream analyses. Cell cycle scores were assigned with the CellCycleScoring function, and regressed out using the ScaleData function. PCA was performed with the RunPCA function, using the previously identified 2,000 most variable genes as input unless specified otherwise (e.g., when using cell cycle marker genes as input). The top genes of each PC were visualised with the VizDimLoadings function, and PCs were plotted against each other using the DimPlot function. Non-linear dimensionality reductions were carried out with PCA inputs using the RunUMAP and RunTSNE functions, and visualised with the DimPlot function.

Differential gene expression was performed with the Seurat function FindMarkers, using the Wilcoxon rank-sum test. Low *p* values were set to a minimum of 2.225 074 × 10^−308^ to permit calculations and plotting on log scales.

LLPS-related proteins and RNAs were identified based on the databases PhaSepDB (http://db.phasep.pro/) and RPS (http://rps.renlab.org), respectively, using all three evidence types (reviewed, high-throughput, and predicted) and filtering by ‘Organism = = Homo sapiens’. The merged list contained 11,202 LLPS-related genes, 8,417 of which were in the 27,716-gene Dendra2 dataframe, and 8,446 were in the 28,442-gene HaloTag dataframe.

Calculation of the Pearson correlation between the expression of the exogenous tag (Dendra2 or HaloTag) and all other genes was carried out using the cor.test function in the stats R package.^84^

#### Parameter estimation and analysis

The feature-barcode matrices containing the cells that passed QC were converted to dense CSV format using mat2csv (10x Genomics Cell Ranger 6.1.2). A Markov chain Monte Carlo (MCMC) sampling approach (100,000 steps) was employed to fit a negative-binomial model (NegativeBinomialModel_no_error.py from https://github.com/mcavallaro/gLoop) to the data as previously described.^29^ Instead of only using 500 cells, the script was modified to use all cells (mean of 3,490 cells per sample) that passed QC (increasing run time). A Bash script was used to parallelise the processs and run the Python script on numerous genes simultaneously, on a server with 128 cores. The means of the posterior samples were used to estimate the mean expression (*μ*), squared coefficient of variation (CV^2^), and burst frequency (k_on_) of each gene. *ν* was calculated from these values using the formula given in the main text, where ${\bar{k}}_{\text{on}}$ is the sample mean burst frequency. The burst size *α*/k_off_ was calculated as (CV^2^_X_ · *μ*_X_) – 1 using the negative binomial approximation as in Cavallaro et al.^29^ Cells with CV^2^ > 2,000 were excluded as these exhibited poor MCMC characteristics such as bad mixing and lack of convergence, based on standard diagnostics, including inspection of the MCMC traces and their autocorrelation functions.

Visually checking the normality of all five estimated and calculated parameters (mean, burst frequency, CV,^2^ *ν*, and burst size) via density and quantile-quantile (Q-Q) plots revealed that none of them are normally distributed, thus requiring non-parametric tests for comparing these across cell lines. This remained the case even when the parameters were log2-transformed to account for heteroscedasticity. Since there are three groups of a categorical independent variable (CTD length) and the dependent variables (the parameters) are quantitative, the Kruskal-Wallis H test was used, which indicated that at least one of the samples was derived from a different distribution to the others. To find out which, pairwise Wilcoxon rank-sum (Mann-Whitney U) tests were conducted (Tables S3–S12). As discussed in the main text, almost all sample pairs (for all parameters) were shown to be statistically different from one another with very low *p* values, presumably due to the interdependencies among gene expression levels. Traditional multiplicity adjustment methods are not suitable since there is only one *p* value for each pairwise comparison, and resampling-based methods commonly used for high-dependence, high-dimensionality data are not appropriate because there is only one value for the kinetic parameter of each gene for each sample.

To assess the impact of Pol II concentration on bursting parameters, we used the model from,^29^ Figure 6. We generated 1,000 combinations of input parameters using Latin hypercube sampling with the following bounds/settings and recorded the resulting bursting dynamics:

0.1≤γ<15,

0.1≤β<15

0.005≤d<0.05,

$$0.001\leq \lambda_{\text{on}}\;<\;2$$

$$0.001\leq \lambda_{off}\;<\;2,$$

δ=1,

l=0.

For the A549 cell parameter estimates, we used the results from,^29^ using the ‘uninfected’ dataset. We selected genes with *μ*_X_ ≥ 0.01 to avoid skewing the distributions with less reliable datapoints.

### Quantification and statistical analysis

Statistical analyses were performed in R. The specific tests used are listed adjacently to reported *p* values in the text and/or figure legend and/or the Methods and/or the figure itself. The number of samples (n) used in each analysis is clear from the main text or is otherwise stated in the Methods or the associated tables.

## Supplemental information

Document S1. Figures S1‒S20 and Tables S1–S13 and S15

Table S14. List of significant differentially expressed genes, related to Figure 7

Data S1. FASTA files of the DNA sequences corresponding to the exogenous RPB1 constructs, related to Figure 1
