# Supplementary material for: N-terminal tagging of RNA Polymerase II shapes transcriptomes more than C-terminal alterations
Source: iScience. 2024 May 7;27(6):109914. doi: 10.1016/j.isci.2024.109914 (PMC11126984; doi:10.1016/j.isci.2024.109914)
Supplement: Document S1. Figures S1‒S20 and Tables S1–S13 and S15 [file mmc1.pdf]

iScience, Volume *27*

Supplemental information

N-terminal tagging of RNA Polymerase II shapes transcriptomes more than C-terminal alterations

Adam Callan-Sidat, Emmanuel Zewdu, Massimo Cavallaro, Juntai Liu, and Daniel Hebenstreit

D25 and H25

1	YSPTSPA	1
2	YEPRSPGG	2
3	YTPQSPS	3
4	YSPTSPS	4
5	YSPTSPS	5
6	YSPTSPN	6
7	YSPTSPS	7
8	YSPTSPS	8
9	YSPTSPS	9
10	YSPTSPS	10
11	YSPTSPS	11
12	YSPTSPS	12
13	YSPTSPS	13
14	YSPTSPS	14
15	YSPTSPS	15
16	YSPTSPS	16
17	YSPTSPS	17
18	YSPTSPS	18
19	YSPTSPS	19
20	YSPTSPS	20
21	YSPTSPS	21
22	YSPTSPKGST	49
23	YSPTSPG	50
24	YSPTSPT	51
25	YSLTSPA	52
	ISPDDSDEEN	

D52 and H52

1	YSPTSPA	1
2	YEPRSPGG	2
3	YTPQSPS	3
4	YSPTSPS	4
5	YSPTSPS	5
6	YSPTSPN	6
7	YSPTSPS	7
8	YSPTSPS	8
9	YSPTSPS	9
10	YSPTSPS	10
11	YSPTSPS	11
12	YSPTSPS	12
13	YSPTSPS	13
14	YSPTSPS	14
15	YSPTSPS	15
16	YSPTSPS	16
17	YSPTSPS	17
18	YSPTSPS	18
19	YSPTSPS	19
20	YSPTSPS	20
21	YSPTSPS	21
22	YSPTSPN	22
23	YSPTSPN	23
24	YTPTSPS	24
25	YSPTSPS	25
26	YSPTSPN	26
27	YTPTSPN	27
28	YSPTSPS	28
29	YSPTSPS	29
30	YSPTSPS	30
31	YSPSSPR	31
32	YTPQSPT	32
33	YTPSSPS	33
34	YSPSSPS	34
35	YSPTSPK	35
36	YTPTSPS	36
37	YSPSSPE	37
38	YTPTSPK	38
39	YSPTSPK	39
40	YSPTSPK	40
41	YSPTSPT	41
42	YSPTTPK	42
43	YSPTSPT	43
44	YSPTSPV	44
45	YTPTSPK	45
46	YSPTSPT	46
47	YSPTSPK	47
48	YSPTSPT	48
49	YSPTSPKGST	49
50	YSPTSPG	50
51	YSPTSPT	51
52	YSLTSPA	52
	ISPDDSDEEN	

D70

1	YSPTSPA	1
2	YEPRSPGG	2
3	YTPQSPS	3
4	YSPTSPS	4
5	YSPTSPS	5
6	YSPTSPN	6
7	YSPTSPS	7
8	YSPTSPS	8
9	YSPTSPS	9
10	YSPTSPS	10
11	YSPTSPS	11
12	YSPTSPS	12
13	YSPTSPS	13
14	YSPTSPS	14
15	YSPTSPS	15
16	YSPTSPS	16
17	YSPTSPS	17
18	YSPTSPS	18
19	YSPTSPS	19
20	YSPTSPS	20
21	YSPTSPS	21
22	YSPTSPN	22
23	YSPTSPN	23
24	YTPTSPS	24
25	YSPTSPS	25
26	YSPTSPN	26
27	YTPTSPN	27
28	YSPTSPS	28
29	YSPTSPS	29
30	YSPTSPS	30
31	YSPSSPR	31
32	YTPQSPT	32
33	YTPSSPS	33
34	YSPSSPS	34
35	YSPTSPK	35
36	YTPTSPS	36
37	YSPSSPE	37
38	YTPTSPK	38
39	YSPTSPK	39
40	YSPTSPK	40
41	YSPTSPT	41
42	YSPTTPK	42
43	YSPTSPT	43
44	YSPTSPV	44
45	YTPTSPK	45
46	YSPTSPT	46
47	YSPTSPK	47
48	YSPTSPT	48
49	YSPTSPKGST	49
50	YSPTSPG	50
51	YSPTSPT	51
52	YTPTSPK	38
53	YSPTSPK	39
54	YSPTSPK	40
55	YSPTSPT	41
56	YSPTTPK	42
57	YSPTSPT	43
58	YSPTSPV	44
59	YTPTSPK	45
60	YSPTSPT	46
61	YSPTSPK	47
62	YSPTSPT	48
63	YSPTSPKGST	49
64	YSPTSPG	50
65	YSPTSPT	51
66	YSLTSPA	52
	ISPDDSDEEN	

H70

1	YSPTSPA	1
2	YEPRSPGG	2
3	YTPQSPS	3
4	YSPTSPS	4
5	YSPTSPS	5
6	YSPTSPN	6
7	YSPTSPS	7
8	YSPTSPS	8
9	YSPTSPS	9
10	YSPTSPS	10
11	YSPTSPS	11
12	YSPTSPS	12
13	YSPTSPS	13
14	YSPTSPS	14
15	YSPTSPS	15
16	YSPTSPS	16
17	YSPTSPS	17
18	YSPTSPS	18
19	YSPTSPS	19
20	YSPTSPS	20
21	YSPTSPS	21
22	YSPTSPN	22
23	YSPTSPN	23
24	YTPTSPS	24
25	YSPTSPS	25
26	YSPTSPN	26
27	YTPTSPN	27
28	YSPTSPS	28
29	YSPTSPS	29
30	YSPTSPS	30
31	YSPSSPR	31
32	YTPQSPT	32
33	YTPSSPS	33
34	YSPSSPS	34
35	YSPTSPK	35
36	YTPTSPS	36
37	YSPSSPE	37
38	YTPTSPK	38
39	YSPTSPK	39
40	YSPTSPK	40
41	YSPTSPT	41
42	YSPTTPK	42
43	YSPTSPT	43
44	YSPTSPV	44
45	YTPTSPK	45
46	YSPTSPT	46
47	YSPTSPK	47
48	YSPTSPT	N/A
49	YSPTTPK	42
50	YSPTSPT	43
51	YSPTSPV	44
52	YTPTSPK	45
53	YSPTSPT	46
54	YSPTSPK	47
55	YSPTSPT	N/A
56	YTPTSPK	38
57	YSPTSPK	39
58	YSPTSPK	40
59	YSPTSPT	41
60	YSPTTPK	42
61	YSPTSPT	43
62	YSPTSPV	44
63	YTPTSPK	45
64	YSPTSPT	46
65	YSPTSPK	47
66	YSPTSPT	48
67	YSPTSPKGST	49
68	YSPTSPG	50
69	YSPTSPT	51
70	YSLTSPA	52
	ISPDDSDEEN	

Figure S1, related to Figure 1 - RPB1 CTD amino acid sequences of the U2OS mutant cell lines. The amino acid sequences of Dendra2-RPB1-25R (D25), Dendra2-RPB1-52R (D52), Dendra2-RPB1-70R (D70), HaloTag -RPB1-25R (H25), HaloTag-RPB1-52R (H52), and HaloTag-RPB1-70R (H70) are shown. Amino acids that match the YSPTSPS consensus repeat sequence are highlighted in red. The position of each repeat in the mutant cell line is numbered

in grey on the left of each sequence, and the repeat in the wildtype human CTD that each repeat originates from is listed in black on the right.

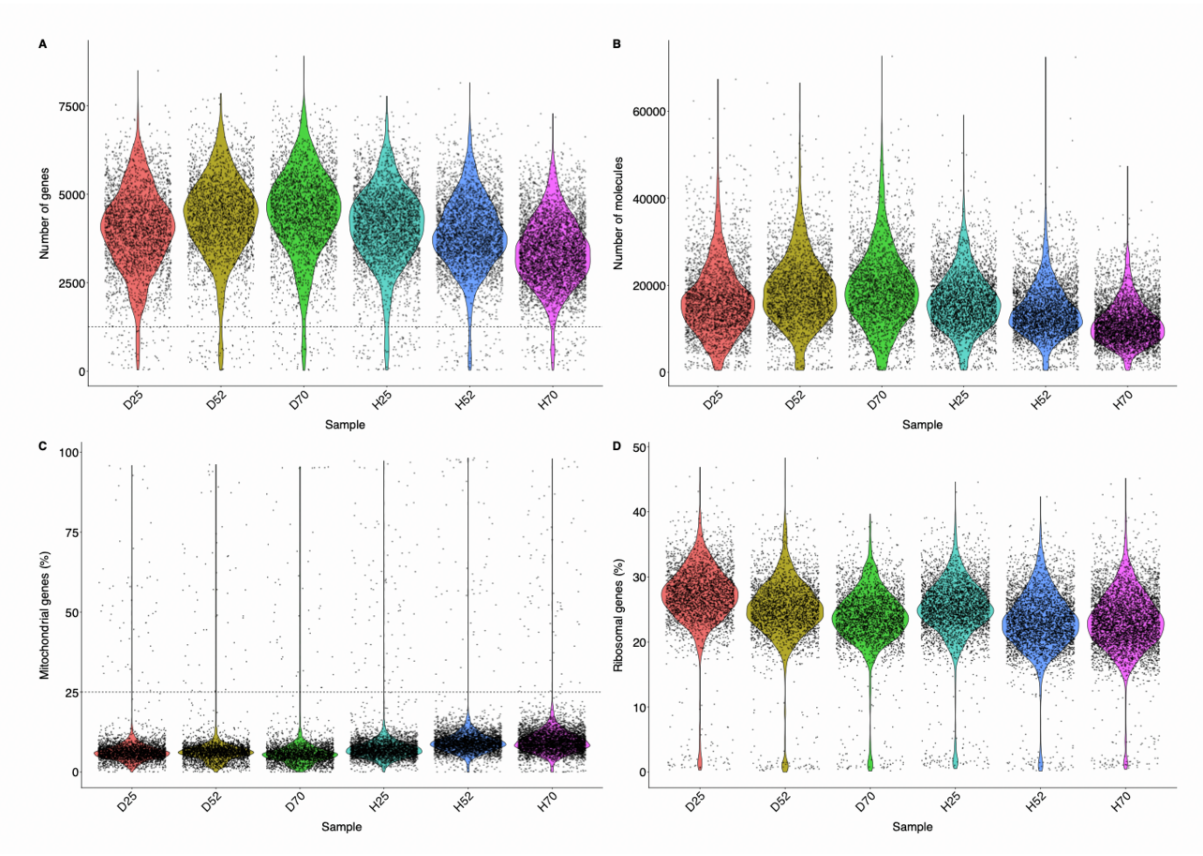

Figure S2, related to Figure 1 - Pre-QC violin plots of scRNA-seq data. **A**, the number of detected genes, **B**, the number of detected molecules, **C**, the percentage of mitochondrial genes, and **D**, the percentage of ribosomal genes, for each cell before QC filtering, for samples Dendra2-RPB1-25R (D25), Dendra2-RPB1-52R (D52), Dendra2-RPB1-70R (D70), HaloTag-RPB1-25R (H25), HaloTag-RPB1-52R (H52), and HaloTag-RPB1-70R (H70).

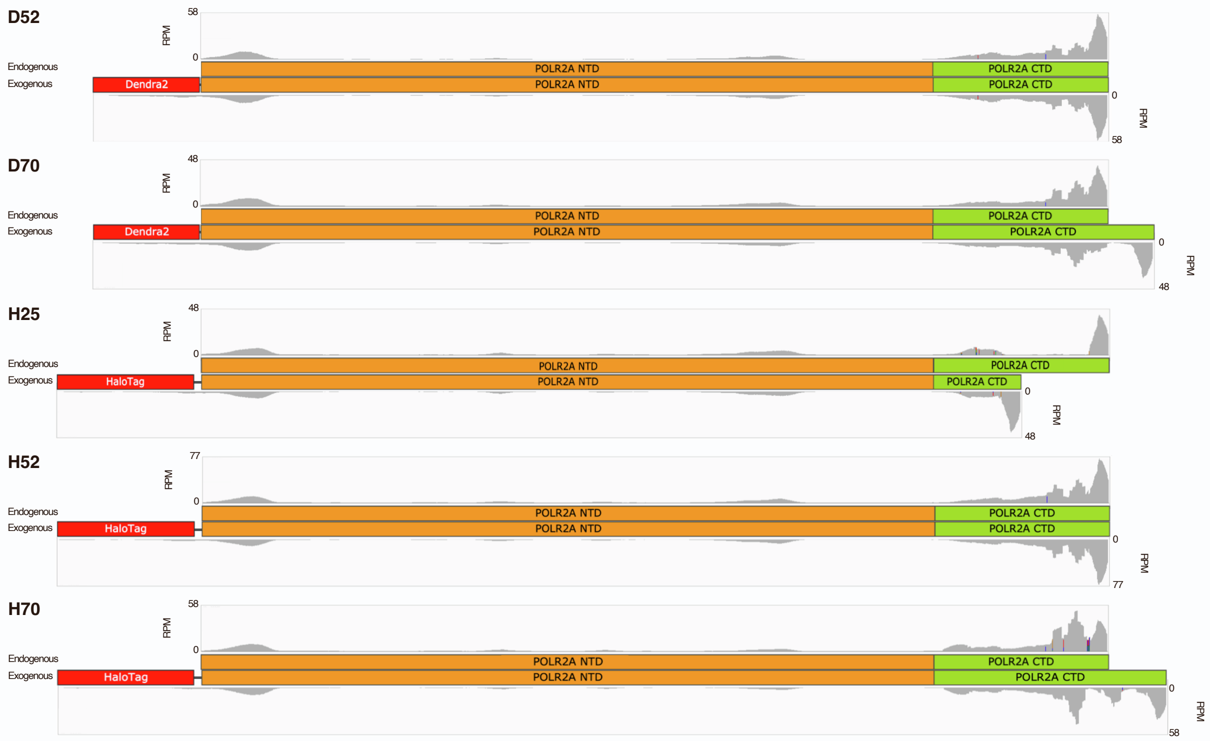

Figure S3, related to Figure 1 - Read coverages at the exo- and endogenous versions of the POL2RA reference sequences (corresponding to RPB1). Shown for all cell lines other than D25 (shown in Fig. 1A). RPM, reads per million total reads. Coverage is computed in 25 bp bins.

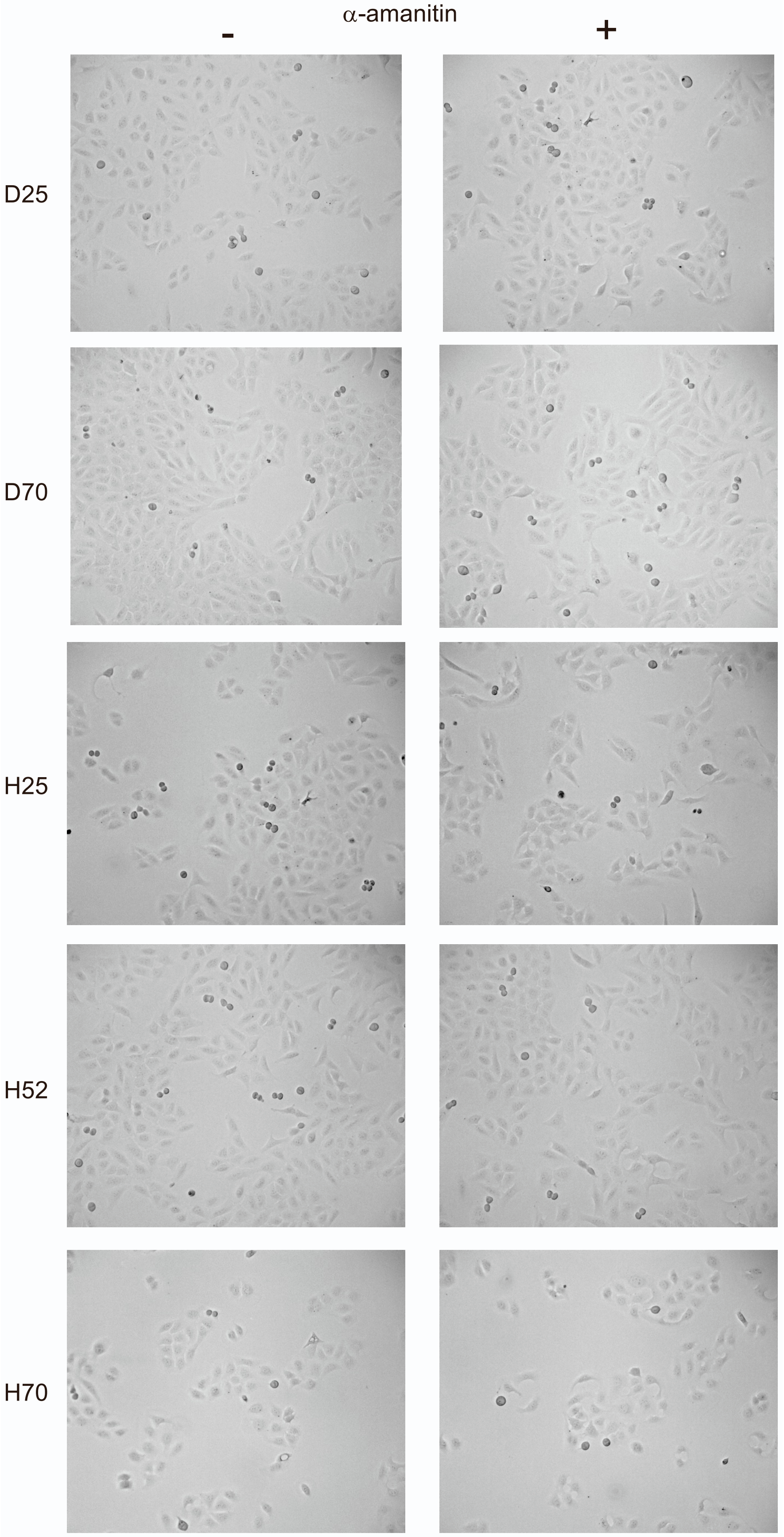

Figure S4, related to Figure 2 - Cells expressing the mutant RPB1 are viable in *α*-amanitin-containing medium. The concentration of *α*-amanitin was 5 μg/ml.

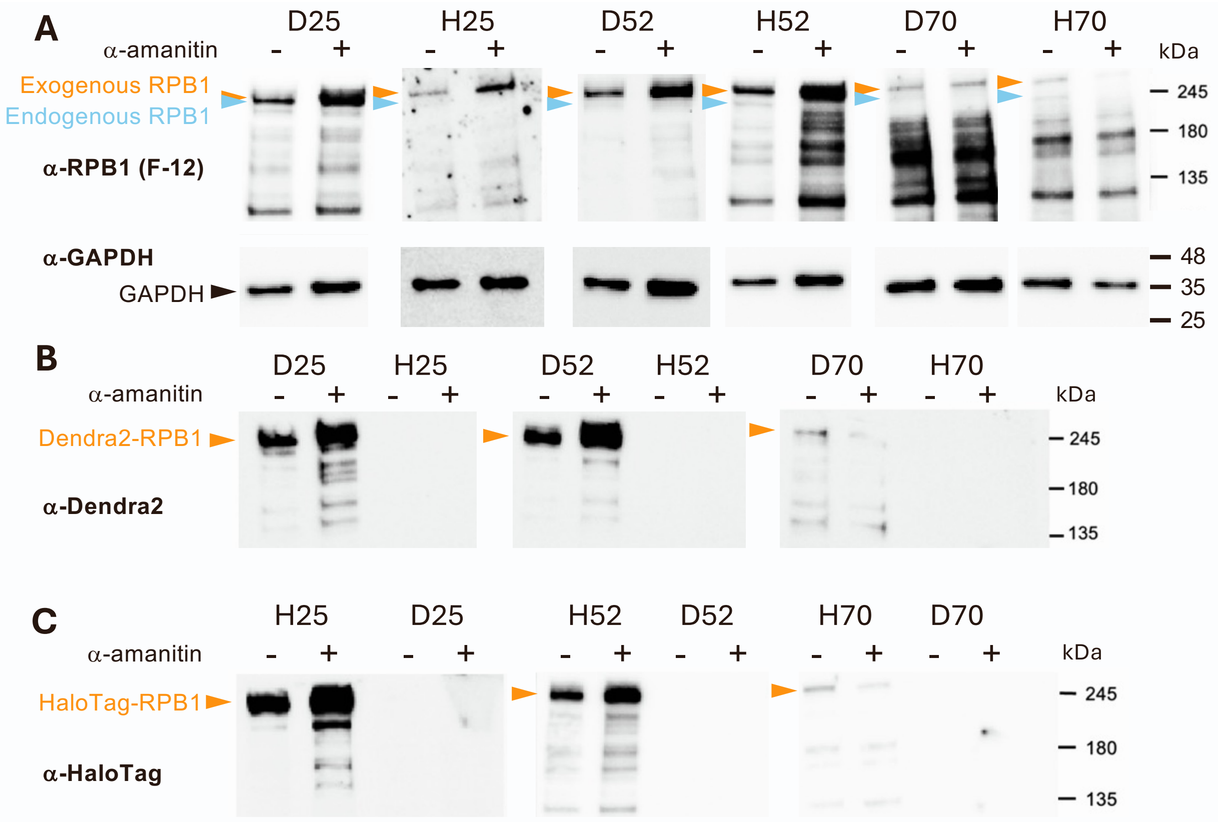

Figure S5, related to Figure 2 - Western blots to detect the various RPB1 variants in the cell lines. Primary antibodies against RPB1 (clone F-12) and GAPDH (**A**), Dendra2 (**B**), and HaloTag (**C**) were used. Cell lines and treatments as indicated. The same lysates were used in B and C. The expected molecular weights are: GAPDH 36 kDa, WT-RPB1 217.2 kDa, Dendra2-RPB1-25R 222.5 kDa, Dendra2-RPB1-52R 243.3 kDa, Dendra2-RPB1-70R 254.1 kDa, HaloTag-RPB1-25R 230.0, HaloTag-RPB1-52R 250.8 kD,a HaloTag-RPB1-70R 264.7 kD.a Note that the WT-RPB1 and Dendra2-RPB1-25R sizes are almost identical and therefore hard to separate.

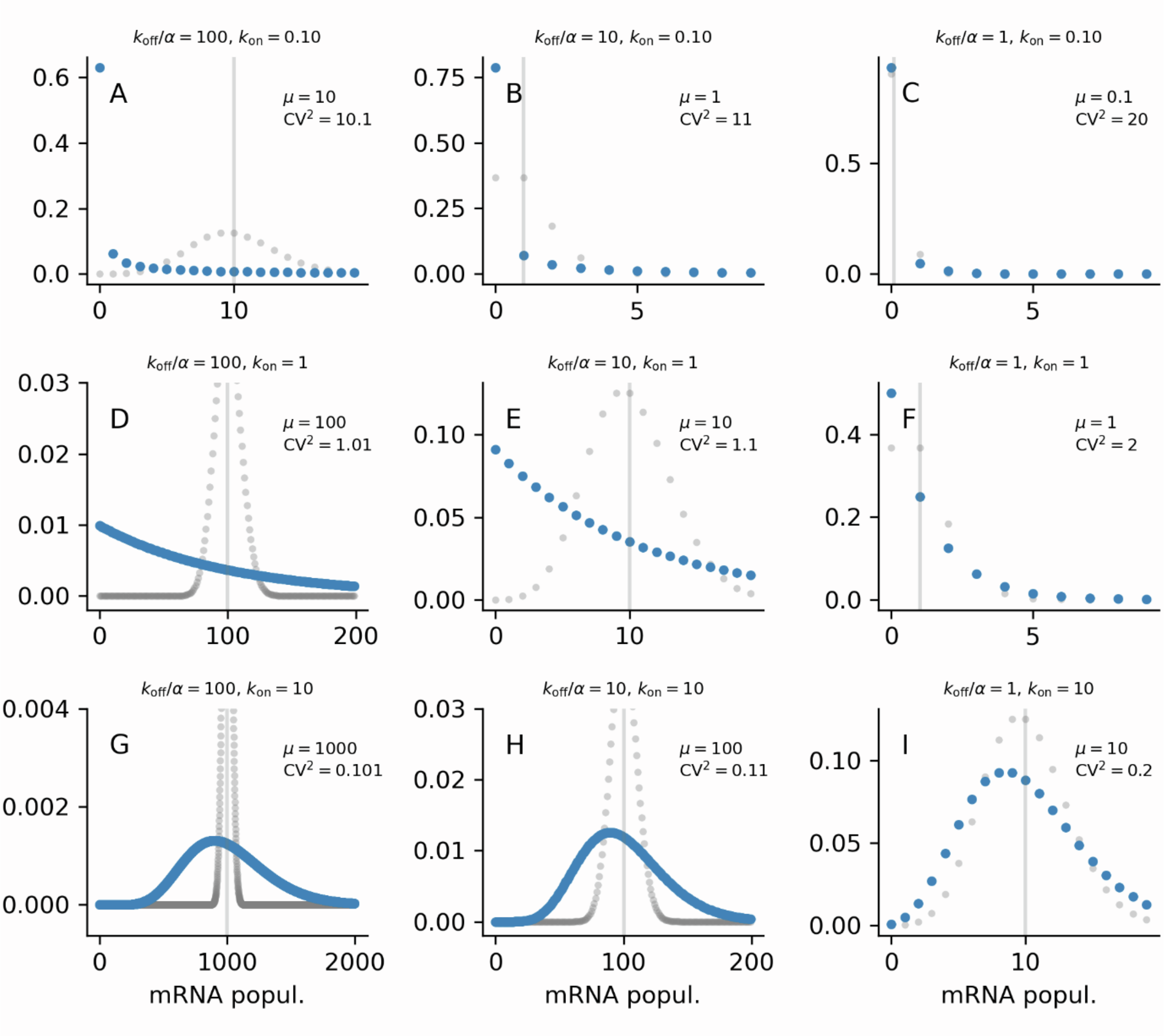

Figure S6, related to Figure 3 - mRNA abundances resulting from different parameter settings. The data was produced from a negative binomial model. The bursting parameter settings are shown on top of each plot. The mRNA probability distributions are shown in blue. The resulting mean and CV^2 are shown inset. Gray dots correspond to Poissonian models (which are the distributions to be expected if no bursting occurs) with identical means (vertical gray lines) as comparison.

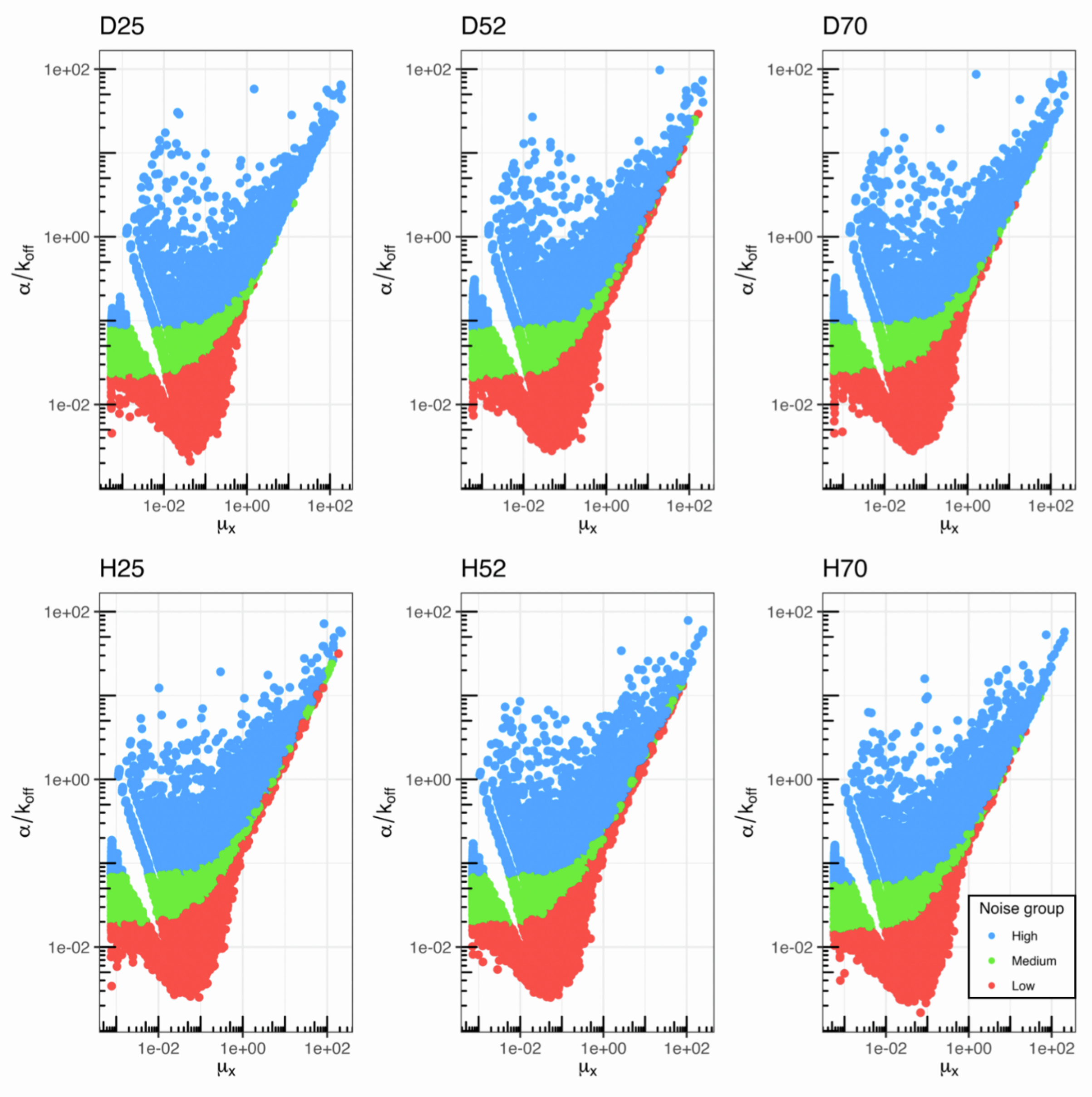

Figure S7, related to Figure 3 - Mean expression (μ_X) vs burst size (α/k_{off}) coloured by noise group. The six samples are mutant U2OS cell lines: Dendra2-RPB1-25R (D25), Dendra2-RPB1-52R (D52), Dendra2-RPB1-70R (D70), HaloTag-RPB1-25R (H25), HaloTag-RPB1-52R (H52), and HaloTag-RPB1-70R (H70).

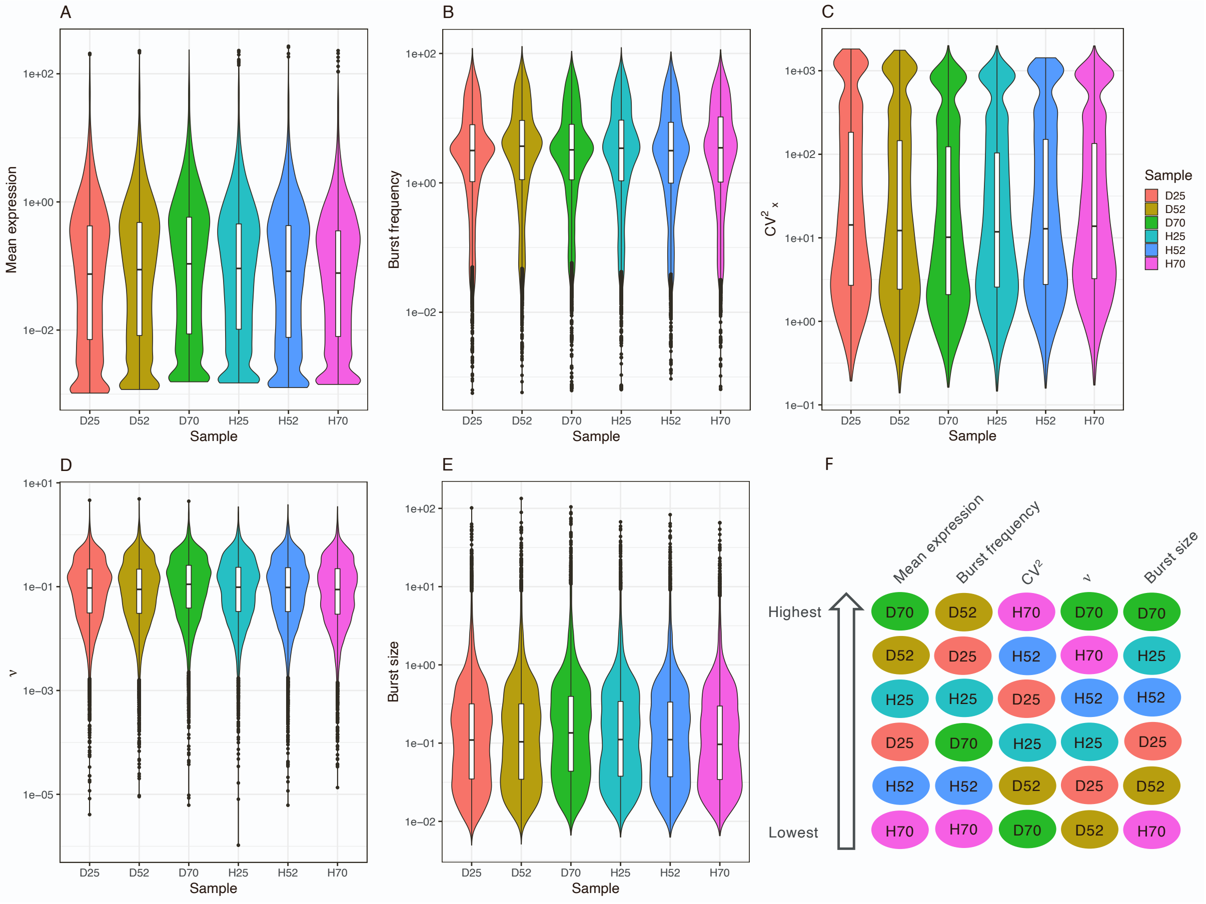

Figure S8, related to Figure 4 - Parameters of transcription of cells with non-zero tag expression. Comparing kinetic parameters estimated from the scRNA-seq data of six mutant U2OS cell lines: Dendra2-RPB1-25R (D25), Dendra2-RPB1-52R (D52), Dendra2-RPB1-70R (D70), HaloTag-RPB1-25R (H25), HaloTag-RPB1-52R (H52), and HaloTag-RPB1-70R (H70). **A-E**, Violin plots with logarithmic y-axes and boxplot overlays. The boxplots indicate Median (central line inside box), 25% and 75% quartiles (lower and upper edge of box, respectively), 1.5 × inter-quartile range (whiskers). **F**, The fifteen pairwise combinations of the six samples were used to compare the kinetic parameters of each gene individually. The sample with the highest number of genes with the highest parameter in each pair was recorded, and the fifteen results were compiled to order the samples.

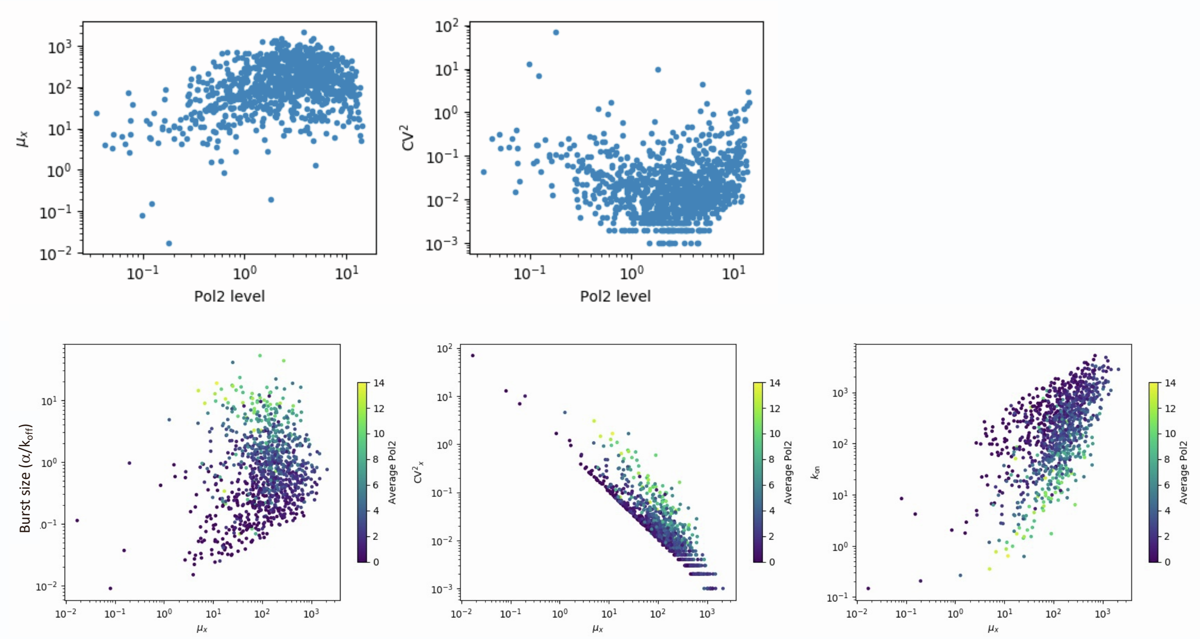

Figure S9, related to Figure 4 - Computational assessment of the effect of Pol2 concentration on transcriptional dynamics. The model used in Cavallaro et al, Genome Biol, 2021, Fig. 6, was explored for the impact of varying Pol2 concentration. Each point in the plots represents a random combination of input model parameters (Materials and Methods). The x-axis (top row) or the colour (bottom row) corresponds to the average Pol2 concentration. The resulting mean mRNA expression (μ_X), noise strengths (CV^2), burst sizes (α/k_{off}), and burst frequencies (k_{on}) are plotted in various combinations as shown. The mean mRNA expression is not affected much by Pol2 concentration.

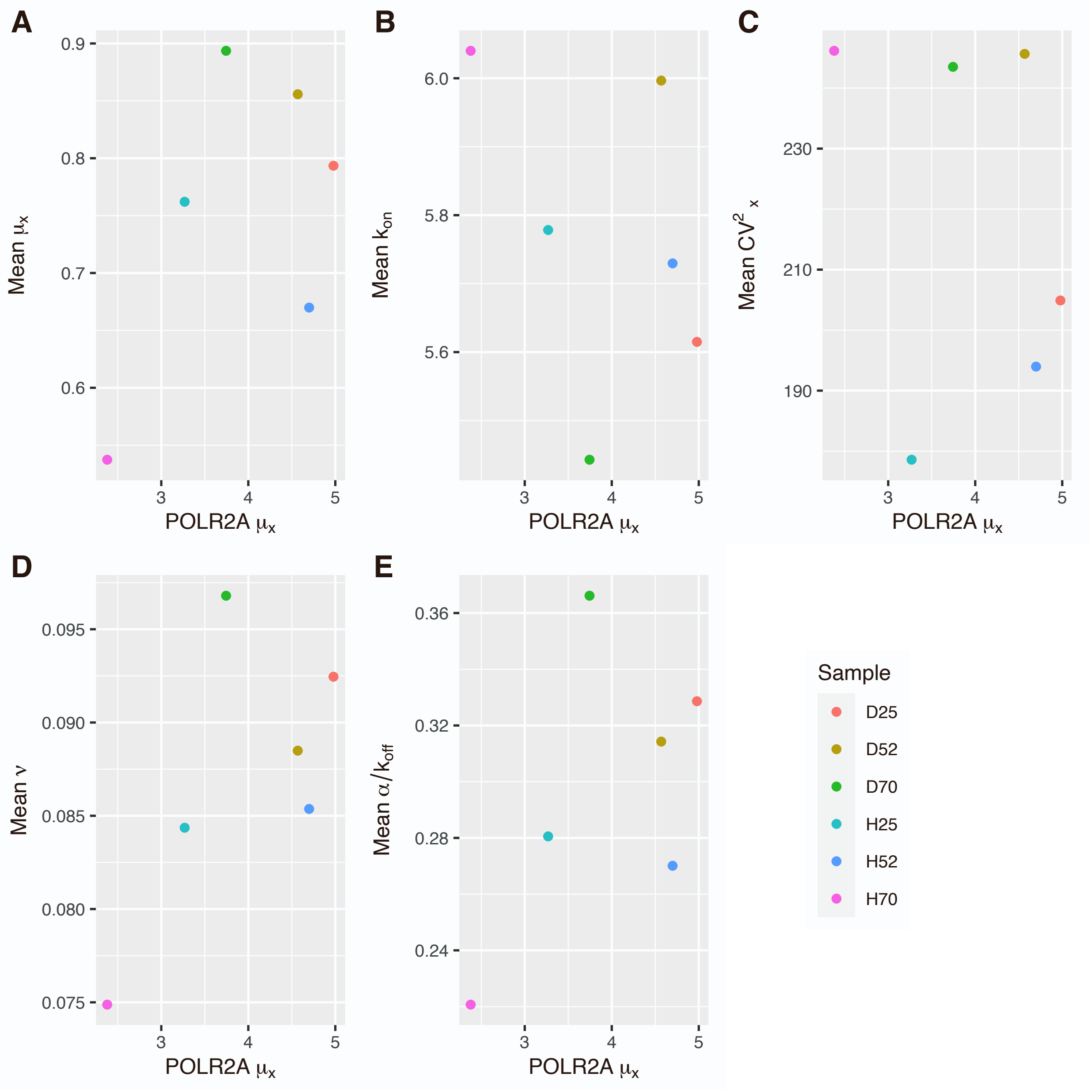

Figure S10, related to Figure 4 - Mean parameter estimates vs mean RPB1 expression levels. Shown for the cell lines as indicated.

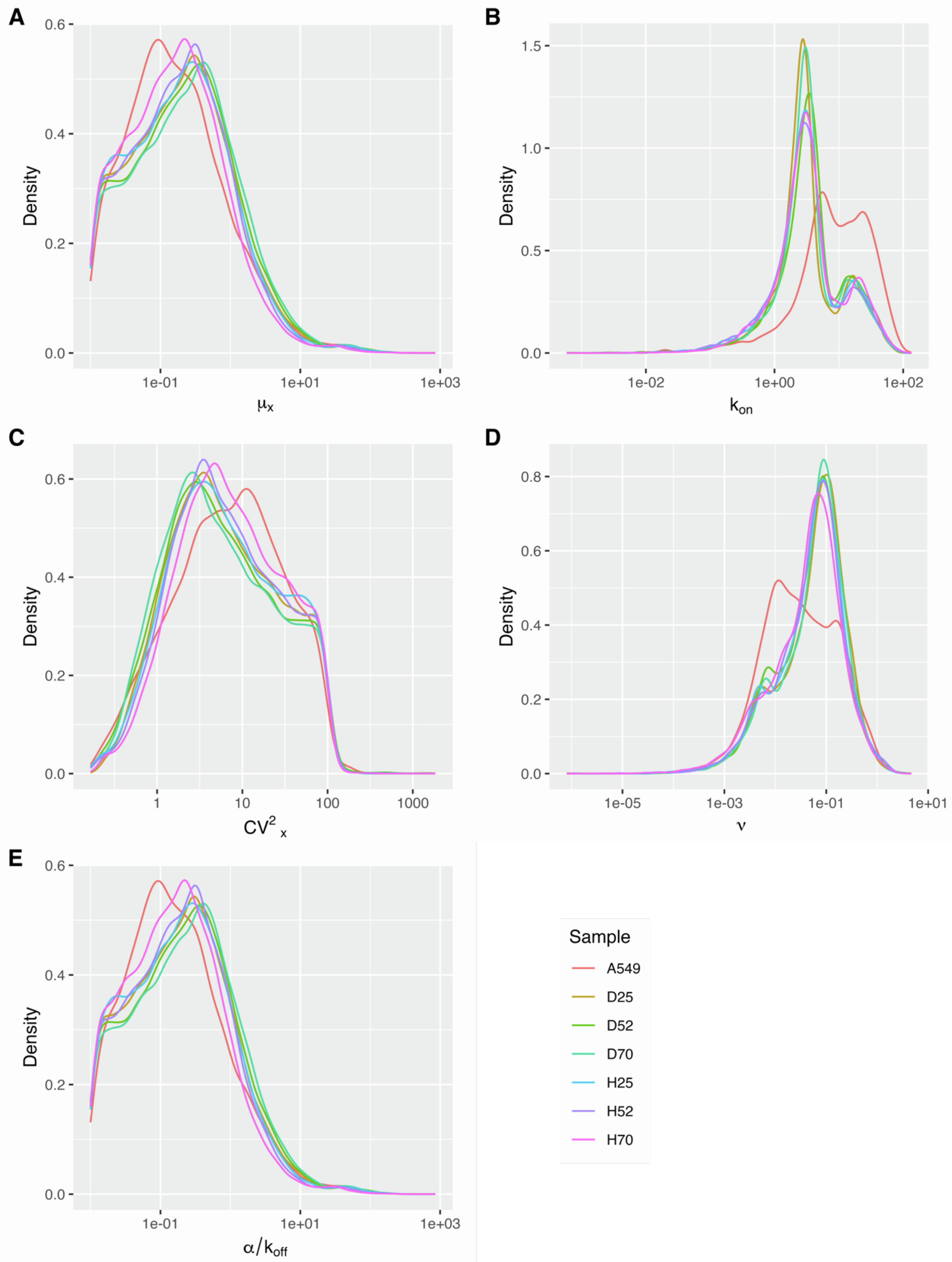

Figure S11, related to Figure 4 - Distributions (kernel density estimates) of bursting parameter estimates for A549 cells compared to the cell lines used in this study.

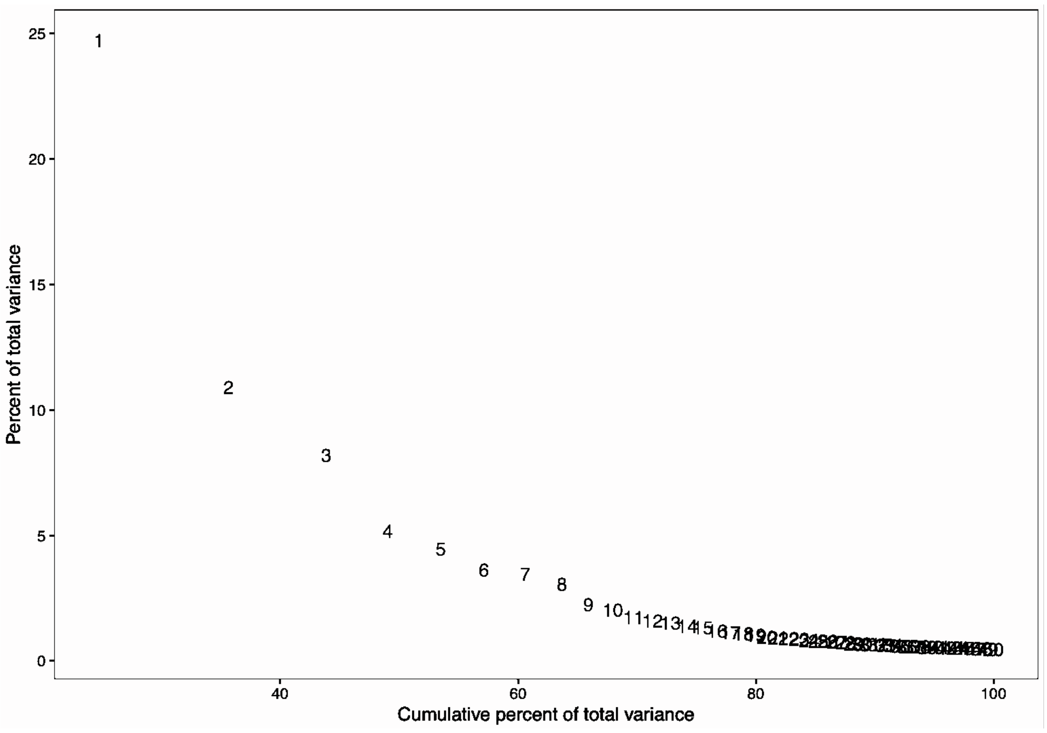

Figure S12, related to Figure 5 - Elbow plot showing the variation each PC contributes to the dataset.

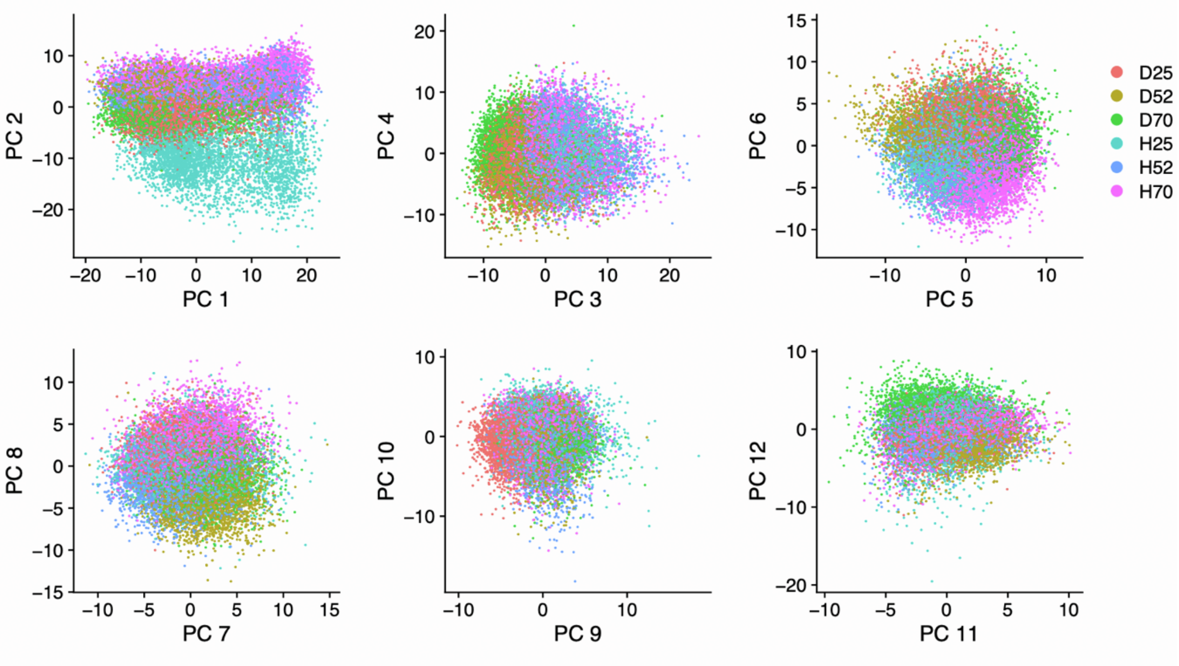

Figure S13, related to Figure 5 - PCA plots of components 1 to 12 representing scRNA-seq data from six U2OS cell lines; Dendra2-RPB1-25R (D25), Dendra2-RPB1-52R (D52), Dendra2-RPB1-70R (D70), HaloTag-RPB1-25R (H25), HaloTag-RPB1-52R (H52), and HaloTag-RPB1-70R (H70).

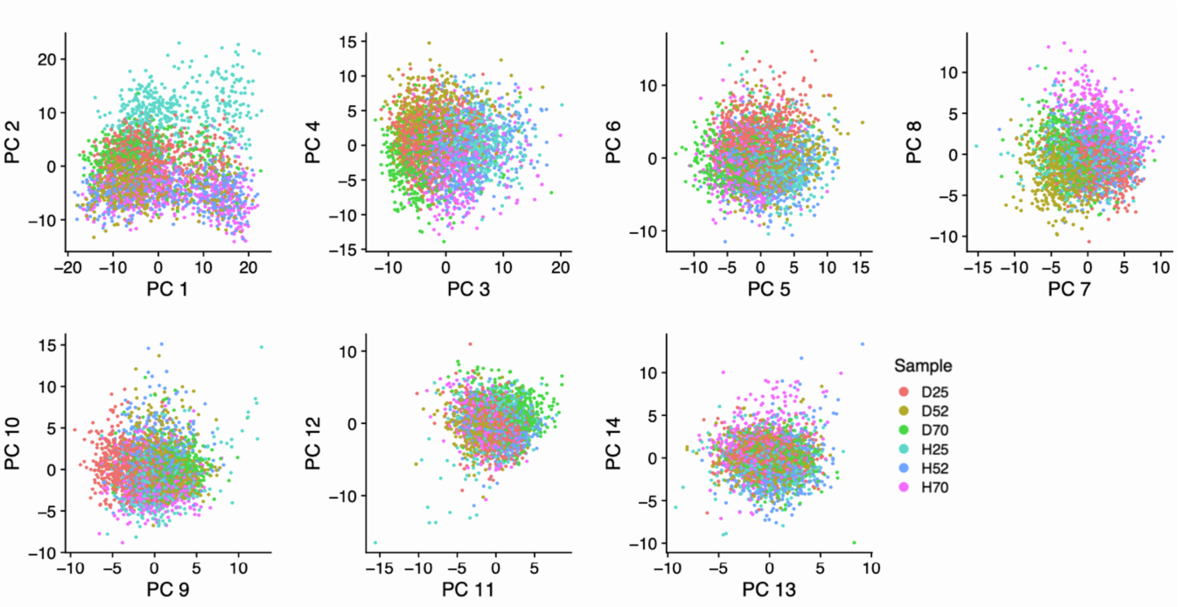

Figure S14, related to Figure 5 - PCA plots of components 1 to 14 representing cells with non-zero tag expression. Based on scRNA-seq data from six U2OS cell lines: Dendra2-RPB1-25R (D25), Dendra2-RPB1-52R (D52), Dendra2-RPB1-70R (D70), HaloTag-RPB1-25R (H25),

HaloTag-RPB1-52R (H52), and HaloTag-RPB1-70R (H70). Only cells with non-zero counts of Dendra2 or HaloTag were used for analysis.

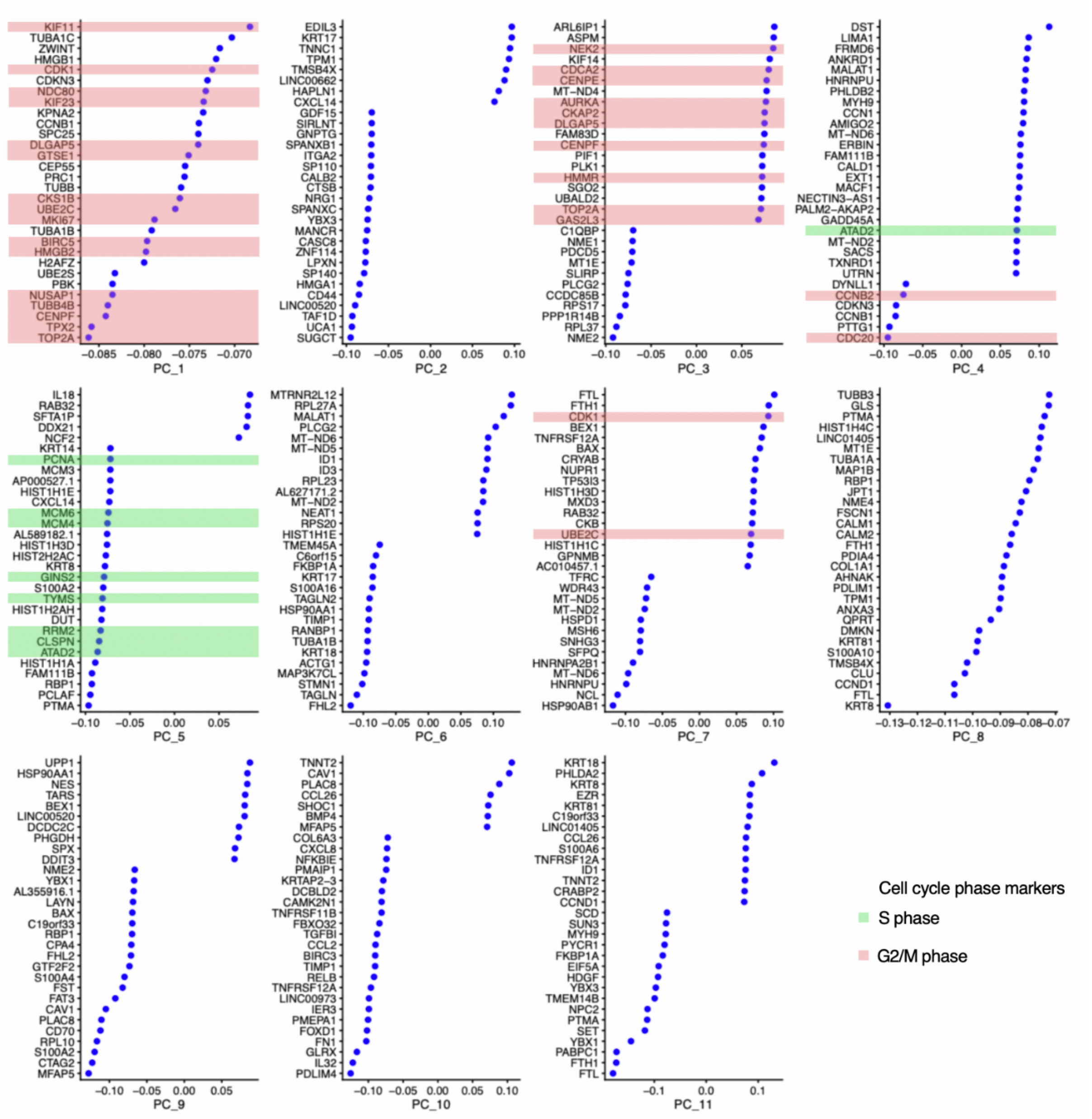

Figure S15, related to Figure 5 - Top genes of significant PCs. Highlighted genes are cell cycle phase markers according to [1] (Table S13).

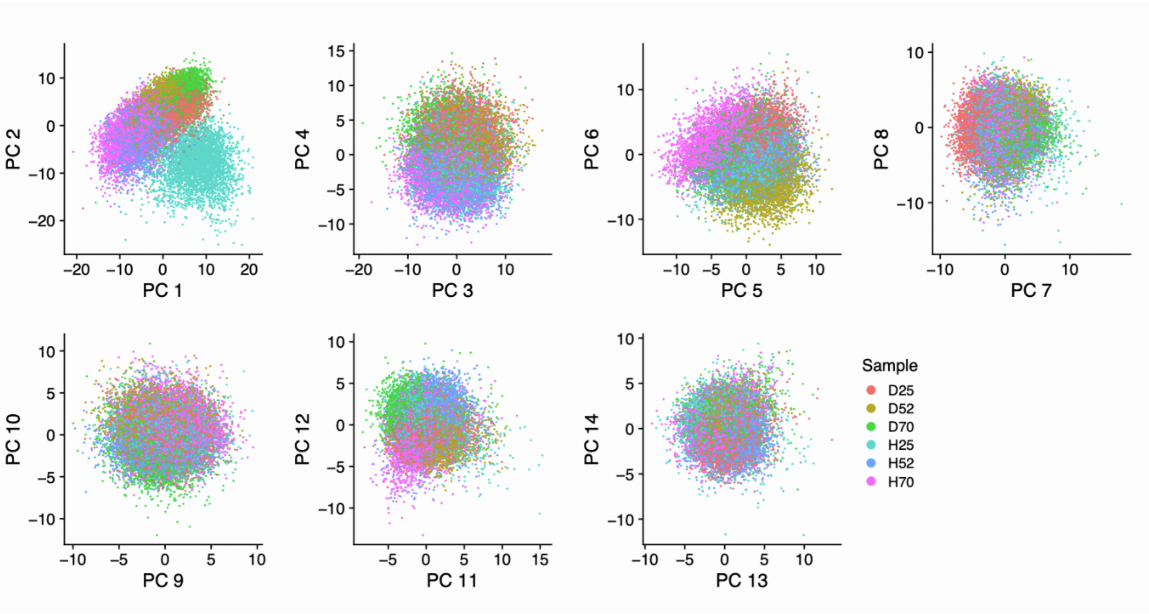

Figure S16, related to Figure 5 - PCA plots after regressing for cell cycle. Representing scRNA-seq data from six U2OS cell lines: Dendra2-RPB1-25R (D25), Dendra2-RPB1-52R (D52), Dendra2-RPB1-70R (D70), HaloTag-RPB1-25R (H25), HaloTag-RPB1-52R (H52), and HaloTag-RPB1-70R (H70).

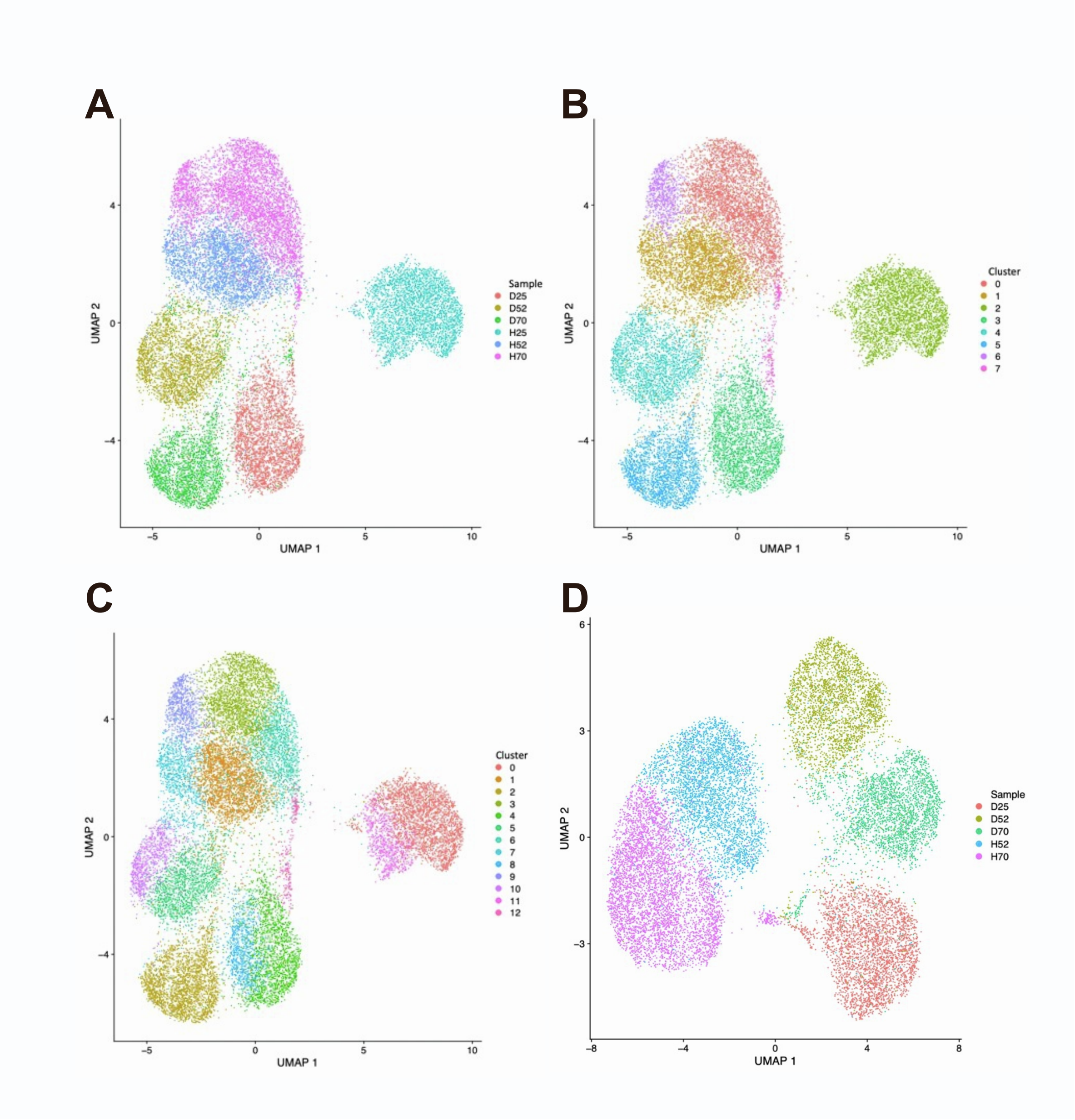

Figure S17, related to Figure 6 - Non-linear dimensionality reduction plots of all QC-passed cells after cell cycle phase regression. Based on scRNA-seq data from six mutant U2OS cell lines: Dendra2-RPB1-25R (D25), Dendra2-RPB1-52R (D52), Dendra2-RPB1-70R (D70), HaloTag-RPB1-25R (H25), HaloTag-RPB1-52R (H52), and HaloTag-RPB1-70R (H70). **A**, UMAP plot coloured by sample. **B**, UMAP plot coloured by cluster, after clustering with a resolution parameter of 0.4. **C**, UMAP plot coloured by cluster, after clustering with a resolution parameter of 1.0. **D**, UMAP plot coloured by sample, excluding H25.

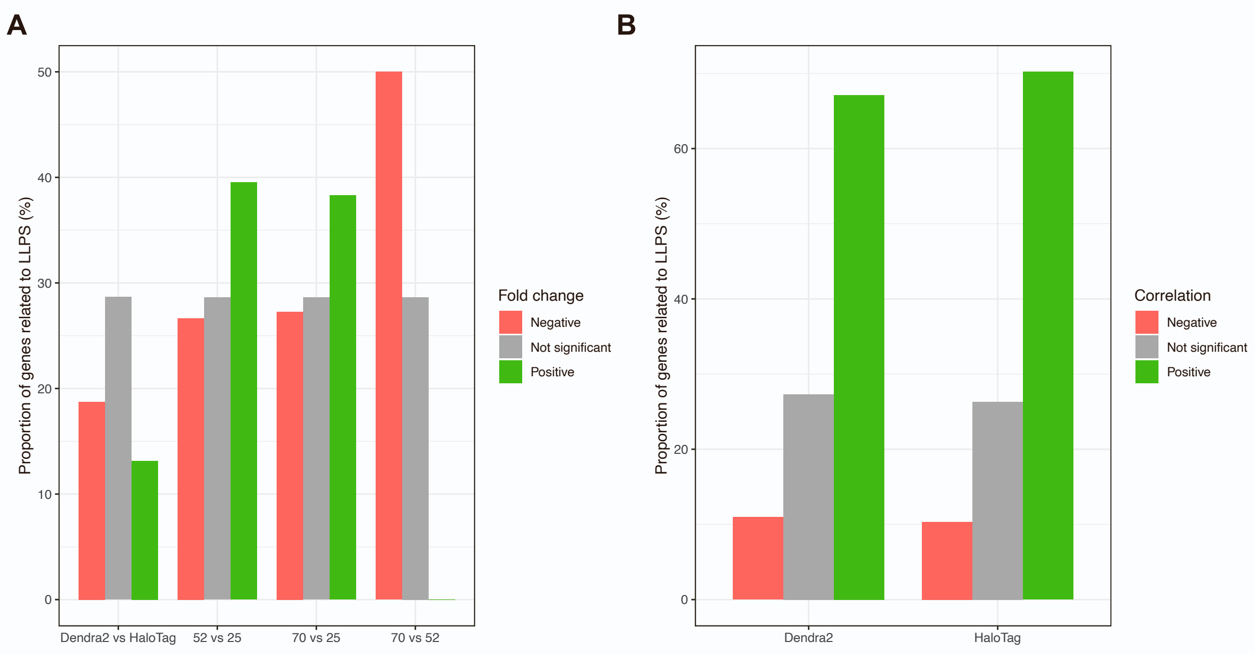

Figure S18, related to Figure 7 - Proportion of LLPS-related genes. Shown for **A**, differentially expressed ones, **B**, tag-expression correlated ones. The proportions of LLPS-related genes among those not significantly differentially expressed or uncorrelated are also shown.

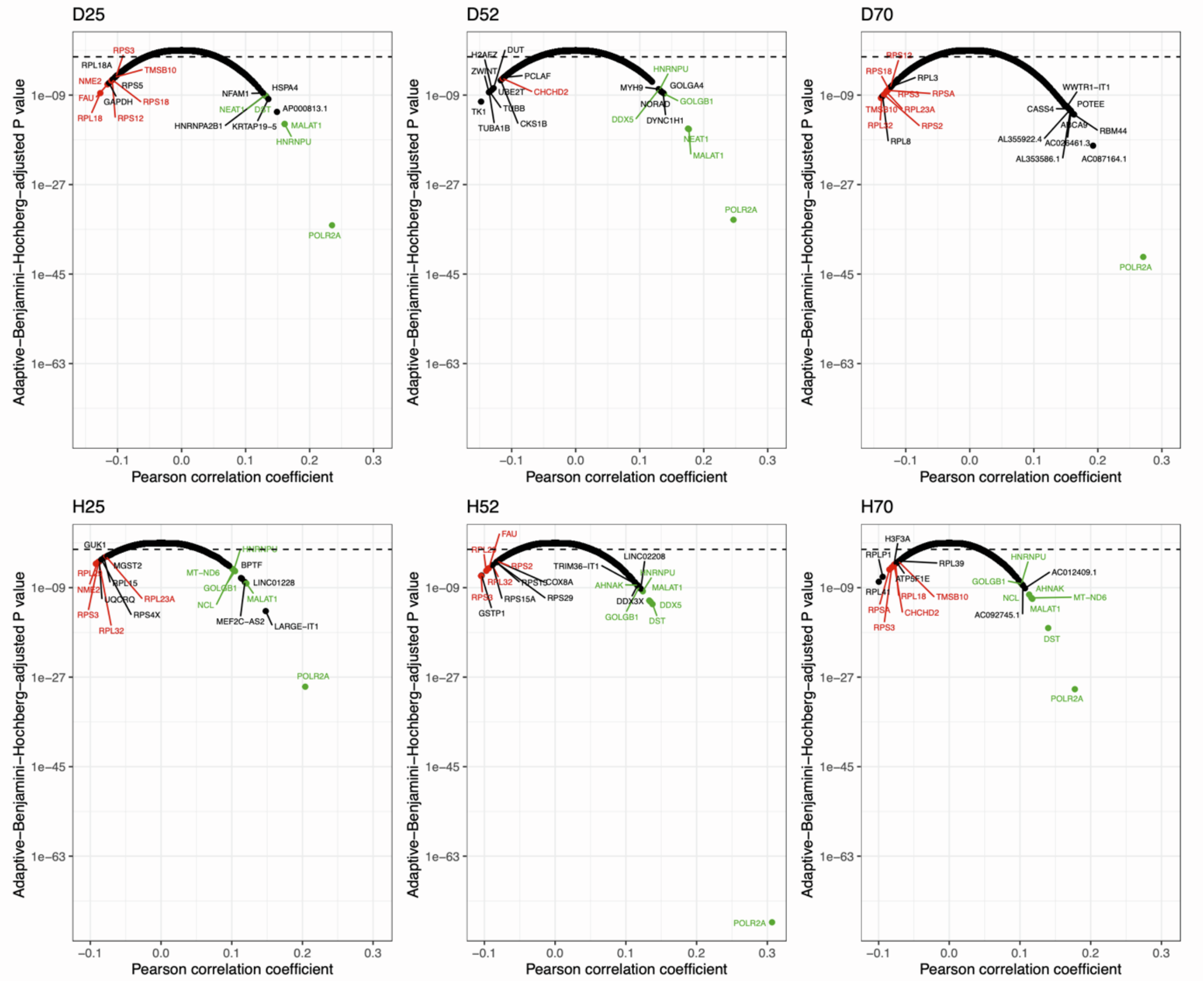

Figure S19, related to Figure 8 – Correlation between exogenous tag expression and gene expression. The linear relationships between Dendra2 expression and the expression of all other genes were evaluated using scRNA-seq data from mutant U2OS cell lines Dendra2-RPB1-25R (D25), Dendra2-RPB1-52R (D52), and Dendra2-RPB1-70R (D70). The same was done with HaloTag expression for cell lines HaloTag-RPB1-25R (H25), HaloTag-RPB1-52R (H52), and HaloTag-RPB1-70R (H70). The Pearson correlation coefficient was plotted against the adaptive Benjamini-Hochberg-adjusted P value. The top ten genes whose expression most negatively correlated with tag expression were labelled, and those in the top ten of more than one sample were coloured red. The same was done with the most positively correlated genes, with the common genes coloured green. The horizontal dashed line is at P value = 0.05.

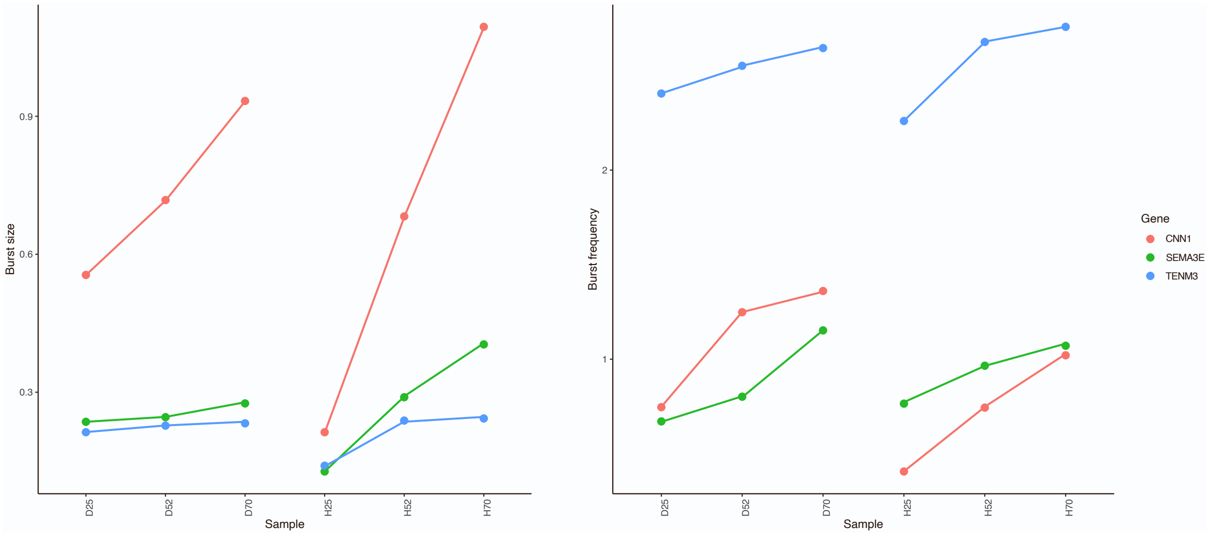

Figure S20, related to Figure 4 - Burst size and frequency scale with CTD length for selected genes. Genes are Calponin 1 (CNN1), Semaphorin 3E (SEMA3E), and Teneurin transmembrane protein 3 (TENM3).

	Pre-subsampling					Post-subsampling			
Sample ID	Number of reads	Number of cells	Mean reads per cell	Median genes per cell	Fraction of reads kept	Number of reads	Number of cells	Mean reads per cell	Median genes per cell
D25	104,133,201	3,185	32,695	4,014	1.000000000	104,133,201	3,185	32,695	4,014
D52	107,943,239	2,820	38,278	4,476	0.964703320	104,132,364	2,812	37,031	4,408
D70	133,587,691	2,791	47,864	5,070	0.779511946	104,136,182	2,777	37,500	4,556
H25	158,994,661	3,608	44,067	5,070	0.654947785	104,126,525	3,592	28,988	4,141
H52	194,632,178	3,804	51,165	5,133	0.535025616	104,120,703	3,786	27,502	3,834
H70	191,743,956	4,814	39,830	4,512	0.543084659	104,124,137	4,790	21,738	3,334

Table S1, related to Figure 1 – Summary statistics before and after subsampling reads to normalise for differences in sequencing depth. For scRNA-seq data from six U2OS cell lines: Dendra2-RPB1-25R (D25), Dendra2-RPB1-52R (D52), Dendra2-RPB1-70R (D70), HaloTag-RPB1-25R (H25), HaloTag-RPB1-52R (H52), and HaloTag-RPB1-70R (H70).

A

Dendra2 (all QC-passed cells)

Sample	count	mean	sd	median	IQR
D25	3106	0.114	0.248	0	0
D52	2738	0.102	0.22	0	0
D70	2701	0.0785	0.204	0	0

P values from Wilcoxon rank sum test		
	D25	D52
D52	4.29E−01	NA
D70	1.81E−07	5.04E−06

HaloTag (all QC-passed cells)

Summary statistics					
Sample	count	mean	sd	median	IQR
H25	3485	0.0675	0.188	0	0
H52	3699	0.0867	0.23	0	0
H70	4687	0.0677	0.215	0	0

P values from Wilcoxon rank sum test		
	H25	H52
H52	2.97E−02	NA
H70	1.08E−03	5.96E−08

GAPDH (all QC-passed cells)

Summary statistics					
Sample	count	mean	sd	median	IQR
D25	3106	3.42	0.328	3.44	0.414
D52	2738	3.46	0.309	3.47	0.399
D70	2701	3.46	0.302	3.47	0.381
H25	3485	3.52	0.324	3.53	0.401
H52	3699	3.54	0.309	3.54	0.393
H70	4687	3.52	0.332	3.53	0.436

P values from Wilcoxon rank sum test					
	D25	D52	D70	H25	H52
D52	1.08E−05	NA	NA	NA	NA
D70	1.62E−05	9.45E−01	NA	NA	NA
H25	2.35E-33	2.00E-13	2.64E-14	NA	NA
H52	7.42E-44	7.80E-20	6.71E-21	0.08735042	NA
H70	3.22E-35	7.77E-14	8.21E-15	0.99398613	0.071828

POLR2A (all QC-passed cells)

Summary statistics					
Sample	count	mean	sd	median	IQR
D25	3106	1.17	0.611	1.22	0.814
D52	2738	1.06	0.545	1.09	0.716
D70	2701	0.894	0.559	0.894	0.744
H25	3485	0.892	0.567	0.899	0.737
H52	3699	1.21	0.651	1.24	0.864
H70	4687	0.906	0.627	0.937	0.835

P values from Wilcoxon rank sum test					
	D25	D52	D70	H25	H52
D52	4.87E−16	NA	NA	NA	NA
D70	4.84E−79	3.02E−32	NA	NA	NA
H25	1.58E-89	4.44E-36	9.17E-01	NA	NA
H52	9.14E-02	1.18E-22	8.55E-94	9.57E-108	NA
H70	1.25E-75	4.29E-25	9.14E-02	4.23E-02	3.64E-94

B

Dendra2 (only QC-passed cells with non-zero expression of Dendra2 or HaloTag)

Summary statistics					
Sample	count	mean	sd	median	IQR
D25	650	0.547	0.239	0.492	0.259
D52	566	0.496	0.201	0.439	0.227
D70	431	0.492	0.239	0.419	0.246

P values from Wilcoxon rank sum test		
	D25	D52
D52	5.19E−05	NA
D70	7.48E−07	1.09E−01

HaloTag (only QC-passed cells with non-zero expression of Dendra2 or HaloTag)

Summary statistics					
Sample	count	mean	sd	median	IQR
H25	454	0.518	0.196	0.471	0.221
H52	531	0.604	0.238	0.565	0.272
H70	471	0.674	0.23	0.636	0.279

P values from Wilcoxon rank sum test		
	H25	H52
H52	1.93E−10	NA
H70	1.63E−30	3.47E−08

GAPDH (only QC-passed cells with non-zero expression of Dendra2 or HaloTag)

Summary statistics					
Sample	count	mean	sd	median	IQR
D25	650	3.37	0.321	3.38	0.409
D52	566	3.42	0.277	3.43	0.378
D70	431	3.42	0.335	3.43	0.346
H25	454	3.47	0.273	3.49	0.32
H52	531	3.48	0.295	3.48	0.364
H70	471	3.45	0.298	3.47	0.394

P values from Wilcoxon rank sum test					
	D25	D52			
D52	1.35E−02	NA	NA	NA	NA
D70	6.23E−03	6.13E−01	NA	NA	NA
H25	1.67E-08	7.60E-04	6.23E-03	NA	NA
H52	1.67E-08	1.25E-03	1.16E-02	0.8703336	NA
H70	6.29E-05	6.35E-02	2.13E-01	0.2132138	0.236526

POLR2A (only QC-passed cells with non-zero expression of Dendra2 or HaloTag)

Summary statistics					
Sample	count	mean	sd	median	IQR
D25	650	1.47	0.554	1.51	0.703
D52	566	1.32	0.535	1.33	0.637
D70	431	1.23	0.597	1.23	0.764
H25	454	1.19	0.601	1.15	0.815
H52	531	1.67	0.696	1.69	0.912
H70	471	1.25	0.659	1.3	0.896

P values from Wilcoxon rank sum test					
	D25	D52			
D52	9.98E−07	NA	NA	NA	NA
D70	9.61E−13	2.18E−03	NA	NA	NA
H25	3.35E-16	2.48E-05	2.60E-01	NA	NA
H52	6.25E-08	1.07E-19	4.09E-24	1.34E-27	NA
H70	8.69E-08	2.22E-01	2.04E-01	1.88E-02	1.97E-19

Table S2, related to Figure 1 - Statistics for cell line comparisons regarding expression of Dendra2, HaloTag, GAPDH and POLR2A. 'count' refers to the numbers of cells, the other

quantities to the normalized expression levels according to scRNA-seq. **A**, all QC-passed cells, corresponding to the results shown in Fig. 1A. **B**, as A, for cells that have non-zero expression of Dendra2 or HaloTag, corresponding to the results shown in Fig. 1B.

Sample pair	W (unpaired)	P value (unpaired)	Estimator (unpaired)	V (paired)	P value (paired)	Estimator (paired)
**D25_D52**	202,289,841	1.80E-11	-1.96E-03	50,990,043	0	-1.73E-02
**D25_D70**	199,812,554	2.89E-13	-2.22E-03	34,974,839	0	-3.36E-02
**D25_H25**	198,069,638	4.00E-09	-1.91E-03	88,102,448	3.94E-67	-3.12E-03
**D25_H52**	204,368,996	3.56E-01	-2.22E-04	118,067,162	1.22E-75	2.86E-03
**D25_H70**	214,736,880	1.91E-24	3.08E-03	165,806,676	0	3.40E-02
**D52_D70**	218,293,484	2.92E-01	-6.10E-05	83,378,143	5.41E-200	-5.31E-03
**D52_H25**	213,238,676	5.13E-01	2.19E-04	128,886,562	5.69E-155	8.24E-03
**D52_H52**	223,238,306	9.69E-09	1.64E-03	168,770,071	0	2.31E-02
**D52_H70**	232,905,611	5.71E-63	6.01E-03	193,188,356	0	6.12E-02
**D70_H25**	212,781,309	1.40E-01	4.26E-04	141,968,578	0	1.91E-02
**D70_H52**	222,341,599	1.78E-10	1.84E-03	170,821,379	0	3.49E-02
**D70_H70**	232,204,294	1.15E-66	6.45E-03	192,589,569	0	7.71E-02
**H25_H52**	216,257,474	1.60E-07	1.56E-03	137,359,190	0	9.17E-03
**H25_H70**	228,294,236	1.74E-60	6.27E-03	177,783,333	0	4.17E-02
**H52_H70**	225,489,596	1.39E-29	3.11E-03	187,946,520	0	3.00E-02

Table S3, related to Figure 4 - Wilcoxon rank-sum test results comparing mean expression between samples. Paired and unpaired Wilcoxon rank-sum tests were performed on the fifteen pairwise combinations of the six samples. W and V are the test statistics, and the estimator is the median difference between the mean expression of a gene from each sample in the pair. Extremely small P values are recorded as 0.

Sample pair	W (unpaired)	P value (unpaired)	Estimator (unpaired)	V (paired)	P value (paired)	Estimator (paired)
**D25_D52**	195,724,871	3.28E-34	-3.18E-01	86,488,967	1.16E-107	-4.07E-01
**D25_D70**	204,461,245	6.81E-04	-7.95E-02	97,518,494	1.23E-15	-1.19E-01
**D25_H25**	197,614,683	3.59E-10	-1.56E-01	90,680,850	7.91E-46	-2.62E-01
**D25_H52**	203,017,186	3.84E-02	-5.02E-02	95,554,281	6.90E-18	-1.51E-01
**D25_H70**	196,352,381	2.84E-08	-1.40E-01	93,418,040	3.10E-22	-1.79E-01
**D52_D70**	230,726,828	2.58E-19	2.28E-01	124,762,885	2.11E-65	2.72E-01
**D52_H25**	218,264,374	1.48E-06	1.28E-01	112,457,876	3.08E-13	1.41E-01
**D52_H52**	227,331,403	1.09E-19	2.41E-01	119,683,951	9.57E-41	2.27E-01
**D52_H70**	219,782,903	3.72E-09	1.59E-01	116,329,984	1.37E-31	2.18E-01
**D70_H25**	206,936,470	7.08E-04	-8.37E-02	99,115,670	5.42E-14	-1.35E-01
**D70_H52**	215,368,169	5.15E-01	1.54E-02	104,437,052	9.06E-04	-5.51E-02
**D70_H70**	208,278,831	8.67E-03	-6.51E-02	102,300,728	5.77E-05	-6.82E-02
**H25_H52**	214,707,730	7.94E-05	1.00E-01	109,903,461	6.84E-09	9.47E-02
**H25_H70**	209,595,828	4.69E-01	1.85E-02	108,261,535	3.96E-06	8.51E-02
**H52_H70**	208,042,085	1.50E-03	-8.09E-02	105,763,255	8.35E-01	-3.00E-03

Table S4, related to Figure 4 - as Table S3, for **burst frequencies**.

Sample pair	W (unpaired)	P value (unpaired)	Estimator (unpaired)	V (paired)	P value (paired)	Estimator (paired)
**D25_D52**	219,086,722	3.22E-13	6.36E-01	139,831,825	0	1.01E+00
**D25_D70**	217,377,743	9.73E-14	6.28E-01	138,375,621	0	9.16E-01
**D25_H25**	212,175,432	1.01E-09	5.47E-01	127,897,630	9.16E-205	1.19E+00
**D25_H52**	206,535,268	3.60E-01	8.35E-02	106,606,668	3.34E-06	9.53E-02
**D25_H70**	191,327,216	6.39E-23	-1.06E+00	62,655,775	0	-2.18E+00
**D52_D70**	220,125,060	6.70E-01	3.34E-02	112,775,592	6.92E-04	4.18E-02
**D52_H25**	211,292,602	3.39E-01	-7.96E-02	105,983,913	7.74E-01	-6.94E-03
**D52_H52**	208,575,722	4.35E-10	-5.61E-01	80,041,582	1.15E-230	-7.44E-01
**D52_H70**	192,387,335	4.63E-63	-1.85E+00	47,212,429	0	-5.00E+00
**D70_H25**	209,376,032	1.75E-01	-1.09E-01	103,716,840	3.50E-02	-5.16E-02
**D70_H52**	206,736,333	1.19E-10	-5.62E-01	83,409,188	1.86E-169	-7.07E-01
**D70_H70**	191,103,477	5.21E-64	-1.82E+00	50,983,918	0	-4.29E+00
**H25_H52**	203,446,907	4.87E-08	-5.01E-01	80,902,617	5.06E-178	-7.95E-01
**H25_H70**	189,409,608	4.52E-59	-1.84E+00	45,769,540	0	-6.24E+00
**H52_H70**	198,756,761	1.49E-27	-1.24E+00	45,603,081	0	-3.95E+00

Table S5, related to Figure 4 - as Table S3, for **CV^2**.

Sample pair	W (unpaired)	P value (unpaired)	Estimator (unpaired)	V (paired)	P value (paired)	Estimator (paired)
**D25_D52**	217,376,778	4.66E-09	2.67E-03	117,431,959	2.61E-47	4.85E-03
**D25_D70**	206,197,262	5.24E-02	-9.36E-04	104,018,760	7.75E-01	-9.02E-05
**D25_H25**	215,046,336	1.24E-17	3.79E-03	115,916,894	1.57E-58	5.47E-03
**D25_H52**	217,089,747	5.45E-23	4.31E-03	116,470,892	4.47E-61	5.55E-03
**D25_H70**	224,534,576	3.24E-77	7.65E-03	131,115,941	1.32E-283	1.22E-02
**D52_D70**	210,153,631	2.44E-14	-3.62E-03	93,996,676	8.08E-73	-5.95E-03
**D52_H25**	216,774,110	3.43E-04	1.57E-03	109,619,492	7.24E-05	1.37E-03
**D52_H52**	221,613,206	1.04E-05	1.92E-03	111,623,733	5.01E-05	1.25E-03
**D52_H70**	228,619,570	8.25E-40	5.31E-03	125,314,710	3.25E-109	6.80E-03
**D70_H25**	223,797,349	1.94E-26	4.90E-03	120,897,161	2.97E-73	6.44E-03
**D70_H52**	229,202,826	2.94E-33	5.46E-03	123,753,939	1.62E-81	6.44E-03
**D70_H70**	236,342,378	4.68E-95	8.86E-03	138,087,256	0.00E+00	1.26E-02
**H25_H52**	211,657,143	1.62E-01	5.80E-04	104,793,870	8.11E-01	-9.08E-05
**H25_H70**	220,732,368	8.01E-24	3.88E-03	118,830,896	6.34E-66	5.38E-03
**H52_H70**	222,407,631	2.36E-18	3.31E-03	124,167,542	2.16E-101	5.83E-03

Table S6, related to Figure 4 - as Table S3, for ***ν***.

Sample pair	W (unpaired)	P value (unpaired)	Estimator (unpaired)	V (paired)	P value (paired)	Estimator (paired)
**D25_D52**	207,646,982	2.42E-02	-1.02E-03	107,889,096	1.40E-03	1.21E-03
**D25_D70**	197,722,449	1.38E-19	-4.46E-03	75,531,701	7.26E-255	-1.47E-02
**D25_H25**	207,564,107	2.87E-02	9.45E-04	114,697,285	1.10E-48	5.85E-03
**D25_H52**	210,079,274	8.74E-05	1.71E-03	120,815,027	1.97E-104	8.94E-03
**D25_H70**	224,641,629	5.83E-78	7.83E-03	146,716,713	0	2.68E-02
**D52_D70**	211,216,110	1.31E-11	-3.35E-03	79,791,971	2.41E-257	-1.59E-02
**D52_H25**	218,717,563	2.11E-07	2.33E-03	117,941,683	8.93E-43	5.87E-03
**D52_H52**	224,131,990	1.01E-10	2.86E-03	128,593,232	9.90E-124	9.27E-03
**D52_H70**	238,581,793	5.19E-102	9.31E-03	157,863,316	0	2.84E-02
**D70_H25**	224,596,379	1.24E-29	5.53E-03	138,980,301	0	2.02E-02
**D70_H52**	230,561,784	2.15E-39	6.40E-03	146,171,663	0	2.27E-02
**D70_H70**	244,823,591	3.01E-169	1.34E-02	168,653,722	0	4.28E-02
**H25_H52**	212,152,614	6.99E-02	7.64E-04	114,019,963	1.65E-26	4.15E-03
**H25_H70**	228,435,899	2.45E-61	6.76E-03	144,171,634	0	2.20E-02
**H52_H70**	229,768,374	7.69E-50	5.87E-03	149,111,581	0	1.95E-02

Table S7, related to Figure 4 - as Table S3, for **burst size**.

Sample pair	W (unpaired)	P value (unpaired)	Estimator (unpaired)	V (paired)	P value (paired)	Estimator (paired)
**D25_D52**	178,784,780	1.89E-10	-2.55E-03	58,347,887	0	-1.45E-02
**D25_D70**	169,005,943	8.36E-22	-5.99E-03	26,818,993	0	-5.48E-02
**D25_H25**	174,800,070	1.43E-07	-2.70E-03	82,029,530	2.42E-27	-3.15E-03
**D25_H52**	180,643,806	1.18E-01	-3.76E-04	102,348,377	9.34E-49	3.87E-03
**D25_H70**	180,100,877	1.68E-20	6.22E-03	136,094,642	0	4.59E-02
**D52_D70**	174,585,172	8.44E-06	-2.55E-03	49,950,616	0	-2.55E-02
**D52_H25**	179,043,079	8.01E-01	2.51E-04	104,768,050	3.01E-93	9.30E-03
**D52_H52**	187,542,852	1.72E-04	2.62E-03	132,494,435	0	2.27E-02
**D52_H70**	187,812,874	1.05E-48	1.21E-02	152,171,860	0	7.45E-02
**D70_H25**	179,797,372	4.06E-09	3.27E-03	126,464,489	0	4.21E-02
**D70_H52**	187,161,133	1.88E-21	6.17E-03	144,528,964	0	5.80E-02
**D70_H70**	188,477,197	1.40E-91	2.01E-02	153,980,519	0	1.21E-01
**H25_H52**	183,449,797	1.71E-06	2.40E-03	110,733,389	1.11E-181	1.10E-02
**H25_H70**	183,849,076	5.15E-54	1.29E-02	139,232,953	0	5.61E-02
**H52_H70**	182,088,602	3.44E-23	7.17E-03	147,425,550	0	4.37E-02

Table S8, related to Figure 4 - as Table S3, **for mean expression of cells with non-zero expression of either tag**.

Sample pair	W (unpaired)	P value (unpaired)	Estimator (unpaired)	V (paired)	P value (paired)	Estimator (paired)
**D25_D52**	174,663,052	3.56E-24	-3.77E-01	81,327,445	1.54E-50	-4.77E-01
**D25_D70**	180,440,913	2.47E-01	3.37E-02	90,271,342	3.79E-01	2.41E-02
**D25_H25**	174,821,652	1.59E-07	-1.79E-01	84,007,332	2.11E-16	-2.77E-01
**D25_H52**	182,551,014	8.36E-01	5.64E-03	90,318,273	2.64E-01	-3.31E-02
**D25_H70**	168,200,843	1.98E-02	-7.62E-02	83,302,112	5.86E-03	-1.03E-01
**D52_D70**	191,321,683	1.72E-29	4.40E-01	101,831,518	8.37E-59	5.17E-01
**D52_H25**	183,593,923	5.64E-06	1.73E-01	94,120,819	2.99E-10	2.26E-01
**D52_H52**	194,275,224	1.92E-23	3.85E-01	102,484,994	9.55E-45	4.43E-01
**D52_H70**	180,119,758	4.91E-13	2.94E-01	94,129,586	1.41E-26	4.09E-01
**D70_H25**	167,149,670	3.10E-10	-2.28E-01	80,490,769	4.93E-18	-3.02E-01
**D70_H52**	176,482,760	5.37E-01	-1.79E-02	87,797,139	2.99E-01	-3.10E-02
**D70_H70**	164,448,884	6.07E-04	-1.21E-01	81,911,983	4.15E-03	-1.02E-01
**H25_H52**	183,971,910	1.30E-07	1.85E-01	95,332,200	2.59E-16	2.69E-01
**H25_H70**	170,611,272	1.56E-02	8.54E-02	87,235,134	1.11E-05	1.71E-01
**H52_H70**	169,513,715	2.25E-02	-7.43E-02	85,149,329	2.79E-01	-3.46E-02

Table S9, related to Figure 4 - as Table S8, for **burst frequency**.

Sample pair	W (unpaired)	P value (unpaired)	Estimator (unpaired)	V (paired)	P value (paired)	Estimator (paired)
**D25_D52**	192,896,753	5.83E-11	4.45E-01	119,584,014	6.24E-262	7.19E-01
**D25_D70**	189,085,842	1.60E-20	5.93E-01	124,949,843	0	1.03E+00
**D25_H25**	185,797,395	4.98E-07	3.44E-01	108,133,731	3.18E-124	6.27E-01
**D25_H52**	183,256,733	3.89E-01	6.08E-02	96,004,683	2.19E-10	1.27E-01
**D25_H70**	160,815,730	1.48E-21	-7.50E-01	60,163,569	1.39E-263	-1.06E+00
**D52_D70**	183,071,907	4.27E-04	2.06E-01	106,019,070	9.65E-105	3.17E-01
**D52_H25**	177,684,178	3.04E-01	-6.53E-02	89,501,840	8.84E-01	2.77E-03
**D52_H52**	177,919,800	2.85E-07	-3.47E-01	73,845,043	6.23E-121	-4.03E-01
**D52_H70**	156,745,997	2.41E-53	-1.20E+00	47,428,902	0	-2.03E+00
**D70_H25**	168,171,656	1.10E-07	-3.16E-01	73,063,745	1.43E-78	-3.56E-01
**D70_H52**	167,071,664	1.36E-21	-6.07E-01	57,682,284	0	-9.49E-01
**D70_H70**	147,992,303	3.42E-86	-1.49E+00	37,236,606	0	-3.03E+00
**H25_H52**	173,170,724	8.93E-07	-3.36E-01	70,865,482	4.15E-132	-5.36E-01
**H25_H70**	152,874,551	2.35E-51	-1.19E+00	45,066,002	0	-2.32E+00
**H52_H70**	161,793,554	1.47E-22	-7.85E-01	51,211,163	0	-1.34E+00

Table S10, related to Figure 4 - as Table S8, for **CV^2**.

Sample pair	W (unpaired)	P value (unpaired)	Estimator (unpaired)	V (paired)	P value (paired)	Estimator (paired)
**D25_D52**	189,223,877	1.44E-03	2.45E-03	95,448,857	8.69E-04	2.05E-03
**D25_D70**	166,854,914	3.34E-31	-1.02E-02	73,306,002	3.13E-104	-1.55E-02
**D25_H25**	177,203,161	2.58E-03	-2.43E-03	85,412,129	2.06E-10	-4.37E-03
**D25_H52**	179,571,911	1.05E-02	-2.04E-03	86,140,950	4.10E-11	-4.46E-03
**D25_H70**	168,600,677	5.24E-02	-1.49E-03	81,613,601	3.67E-07	-3.63E-03
**D52_D70**	163,913,515	1.53E-47	-1.26E-02	71,012,272	1.03E-135	-1.78E-02
**D52_H25**	172,341,846	1.36E-09	-4.81E-03	82,064,197	1.63E-22	-6.81E-03
**D52_H52**	177,206,856	6.95E-09	-4.56E-03	82,933,407	1.18E-30	-7.29E-03
**D52_H70**	167,157,938	1.12E-07	-4.04E-03	79,192,963	1.73E-22	-6.53E-03
**D70_H25**	182,515,793	1.92E-17	7.64E-03	96,846,761	3.43E-42	1.09E-02
**D70_H52**	186,202,659	7.80E-18	7.68E-03	97,782,430	4.53E-35	9.25E-03
**D70_H70**	177,305,486	1.94E-20	8.01E-03	92,961,126	3.47E-36	1.02E-02
**H25_H52**	178,758,550	7.20E-01	2.88E-04	87,838,135	7.04E-02	-1.27E-03
**H25_H70**	169,292,193	2.64E-01	8.87E-04	84,049,537	9.61E-01	-2.64E-05
**H52_H70**	172,304,656	6.70E-01	3.34E-04	87,368,454	4.96E-02	1.34E-03

Table S11, related to Figure 4 - as Table S8, for ***v***.

Sample pair	W (unpaired)	P value (unpaired)	Estimator (unpaired)	V (paired)	P value (paired)	Estimator (paired)
**D25_D52**	186,988,551	2.54E-01	7.81E-04	96,849,841	2.70E-07	3.83E-03
**D25_D70**	161,908,099	1.62E-59	-1.35E-02	63,188,091	1.96E-270	-3.41E-02
**D25_H25**	175,264,263	1.38E-06	-3.42E-03	90,137,952	9.17E-01	-8.23E-05
**D25_H52**	178,405,588	2.71E-04	-2.60E-03	91,945,273	3.08E-01	8.35E-04
**D25_H70**	172,114,888	1.37E-01	1.02E-03	96,184,113	5.44E-51	1.47E-02
**D52_D70**	160,882,370	2.67E-67	-1.43E-02	60,228,662	0	-3.91E-02
**D52_H25**	172,400,602	1.91E-09	-4.18E-03	85,894,377	3.16E-06	-3.85E-03
**D52_H52**	178,128,823	7.80E-07	-3.46E-03	89,261,485	1.19E-03	-2.41E-03
**D52_H70**	172,720,741	9.42E-01	4.59E-05	96,231,757	8.54E-42	1.21E-02
**D70_H25**	185,877,505	8.46E-32	9.85E-03	108,694,685	2.29E-194	3.35E-02
**D70_H52**	190,331,490	5.88E-36	1.04E-02	111,303,936	3.10E-204	3.26E-02
**D70_H70**	186,115,420	3.73E-72	1.48E-02	112,701,035	0	5.01E-02
**H25_H52**	179,580,504	2.57E-01	8.12E-04	90,369,146	1.17E-01	1.32E-03
**H25_H70**	174,874,848	3.51E-11	4.57E-03	96,841,293	7.96E-71	1.71E-02
**H52_H70**	177,103,570	3.73E-07	3.52E-03	101,003,046	6.13E-95	1.90E-02

Table S12, related to Figure 4 - as Table S8, for **burst size**.

S phase					
MCM5	PCNA	TYMS	FEN1	MCM2	MCM4
RRM1	UNG	GINS2	MCM6	CDCA7	DTL
PRIM1	UHRF1	MLF1IP	HELLS	RFC2	RPA2
NASP	RAD51AP1	GMNN	WDR76	SLBP	CCNE2
UBR7	POLD3	MSH2	ATAD2	RAD51	RRM2
CDC45	CDC6	EXO1	TIPIN	DSCC1	BLM
CASP8AP2	USP1	CLSPN	POLA1	CHAF1B	BRIP1
E2F8					

G2/M phase					
HMGB2	CDK1	NUSAP1	UBE2C	BIRC5	TPX2
TOP2A	NDC80	CKS2	NUF2	CKS1B	MKI67
TMPO	CENPF	TACC3	FAM64A	SMC4	CCNB2
CKAP2L	CKAP2	AURKB	BUB1	KIF11	ANP32E
TUBB4B	GTSE1	KIF20B	HJURP	CDCA3	HN1
CDC20	TTK	CDC25C	KIF2C	RANGAP1	NCAPD2
DLGAP5	CDCA2	CDCA8	ECT2	KIF23	HMMR
AURKA	PSRC1	ANLN	LBR	CKAP5	CENPE
CTCF	NEK2	G2E3	GAS2L3	CBX5	CENPA

Table S13, related to Figure 5 - Cell cycle marker genes. From [S1].

Comparison	Fold change	LLPS-related	Number	Percent
Dendra2 vs HaloTag	Negative	TRUE	6	18.8
Dendra2 vs HaloTag	Negative	FALSE	26	81.3
Dendra2 vs HaloTag	Positive	TRUE	5	13.2
Dendra2 vs HaloTag	Positive	FALSE	33	86.8
52 vs 25	Negative	TRUE	8	26.7
52 vs 25	Negative	FALSE	22	73.3
52 vs 25	Positive	TRUE	17	39.5
52 vs 25	Positive	FALSE	26	60.5
70 vs 52	Negative	TRUE	3	50.0
70 vs 52	Negative	FALSE	3	50.0
70 vs 52	Positive	TRUE	0	0.0
70 vs 52	Positive	FALSE	9	100.0
70 vs 25	Negative	TRUE	9	27.3
70 vs 25	Negative	FALSE	24	72.7
70 vs 25	Positive	TRUE	18	38.3
70 vs 25	Positive	FALSE	29	61.7
Dendra2 vs HaloTag	Not significant	TRUE	8520	28.7
Dendra2 vs HaloTag	Not significant	FALSE	21204	71.3
52 vs 25	Not significant	TRUE	8506	28.6
52 vs 25	Not significant	FALSE	21215	71.4
70 vs 52	Not significant	TRUE	8528	28.6
70 vs 52	Not significant	FALSE	21251	71.4
70 vs 25	Not significant	TRUE	8504	28.6
70 vs 25	Not significant	FALSE	21210	71.4

Tag	Correlation	LLPS-related	Number	Percent
Dendra2	Negative	TRUE	84	11.0
Dendra2	Negative	FALSE	682	89.0
Dendra2	Positive	TRUE	1642	67.1
Dendra2	Positive	FALSE	805	32.9
Dendra2	Not significant	TRUE	6691	27.3
Dendra2	Not significant	FALSE	17812	72.7
HaloTag	Negative	TRUE	42	10.3
HaloTag	Negative	FALSE	365	89.7
HaloTag	Positive	TRUE	1638	70.2
HaloTag	Positive	FALSE	695	29.8
HaloTag	Not significant	TRUE	6766	26.3
HaloTag	Not significant	FALSE	18936	73.7

Table S15, related to Figure 7 - Numbers of LLPS-related genes among differentially expressed and tag-correlated ones. The rest of the genes is included as 'Not significant'.

SUPPLEMENTAL REFERENCES

S1. Tirosh, I., Izar, B., Prakadan, S.M., Wadsworth, M.H., 2nd, Treacy, D., Trombetta, J.J., Rotem, A., Rodman, C., Lian, C., Murphy, G., et al. (2016). Dissecting the multicellular ecosystem of metastatic melanoma by single-cell RNA-seq. Science *352*, 189-196. 10.1126/science.aad0501.
